# Single molecule/particle tracking analysis program SMTracker 2.0 reveals different dynamics of proteins within the RNA degradosome complex in *Bacillus subtilis*

**DOI:** 10.1093/nar/gkab696

**Published:** 2021-08-20

**Authors:** Luis M Oviedo-Bocanegra, Rebecca Hinrichs, Daniel Andreas Orlando Rotter, Simon Dersch, Peter L Graumann

**Affiliations:** Centre for Synthetic Microbiology (SYNMIKRO) and Fachbereich Chemie, Philipps-Universität Marburg, 35032 Marburg, Germany; Centre for Synthetic Microbiology (SYNMIKRO) and Fachbereich Chemie, Philipps-Universität Marburg, 35032 Marburg, Germany; Centre for Synthetic Microbiology (SYNMIKRO) and Fachbereich Chemie, Philipps-Universität Marburg, 35032 Marburg, Germany; Centre for Synthetic Microbiology (SYNMIKRO) and Fachbereich Chemie, Philipps-Universität Marburg, 35032 Marburg, Germany; Centre for Synthetic Microbiology (SYNMIKRO) and Fachbereich Chemie, Philipps-Universität Marburg, 35032 Marburg, Germany

## Abstract

Single-molecule (particle) tracking is a powerful method to study dynamic processes in cells at highest possible spatial and temporal resolution. We have developed SMTracker, a graphical user interface for automatic quantifying, visualizing and managing of data. Version 2.0 determines distributions of positional displacements in *x-* and *y-*direction using multi-state diffusion models, discriminates between Brownian, sub- or superdiffusive behaviour, and locates slow or fast diffusing populations in a standardized cell. Using SMTracker, we show that the *Bacillus subtilis* RNA degradosome consists of a highly dynamic complex of RNase Y and binding partners. We found similar changes in molecule dynamics for RNase Y, CshA, PNPase and enolase, but not for phosphofructokinase, RNase J1 and J2, to inhibition of transcription. However, the absence of PfkA or of RNase J2 affected molecule dynamics of RNase Y-mVenus, indicating that these two proteins are indeed part of the degradosome. Molecule counting suggests that RNase Y is present as a dimer in cells, at an average copy number of about 500, of which 46% are present in a slow-diffusive state and thus likely engaged within degradosomes. Thus, RNase Y, CshA, PNPase and enolase likely play central roles, and RNase J1, J2 and PfkA more peripheral roles, in degradosome architecture.

## INTRODUCTION

Degradation and processing of RNA is a central aspect within the central dogma in biology. The regulation of the lifetime of mRNAs, the specific cleavage of mRNAs to yield differentially regulated translation events, and the maturation of stable RNAs are essential for cellular life (1–3). In many bacteria, RNase E plays a key role in RNA processing, and in the initiation of degradation events. RNase E contains an amphipathic helix at its N-terminus and thereby associates with the cell membrane, where it forms a complex with several additional proteins, the so called RNA degradosome (4). Present within this complex are one exonuclease (PNPase) that after initial endonucleolytic cleavage by RNase E takes over processive degradation of mRNA, an RNA helicase that can open up secondary structures in RNA, as well as one enzymes thought to act as moonlighting protein: enolase is a glycolytic enzyme, and appears to help in complex formation, which is proposed to be centred around RNase E.

Many bacteria, including Gram positives lack RNase E, but contain an analogous enzyme, RNase Y, which associates with the membrane through a predicted transmembrane helix at its N-terminus (5). Like RNase E, it is thought to be the central component of an RNA degradosome, containing RNases J1 and J2, PNPase, RNA helicase CshA, as well as enolase and phosphofructokinase I (PfkA), also a glycolytic enzyme (6,7). Evidence for this idea stems from two-hybrid experiments and interaction studies after chemical crosslinking. Interestingly, RNase Y has been proposed to act within a second complex, the so-called Y-complex (or RicAFT), which contains three additional soluble proteins, and has been shown to interact with RNase Y (8). Lack of Y-complex proteins leads to loss of function of riboswitches as well as to dysregulation of global mRNA processing, analogous to loss of RNase Y (9). Interestingly, RNase E as well as RNase Y have been shown to forms clusters underneath the cell membrane, which in case of RNase E are dynamic and short-lived (10–13).

We sought to further study the putative RNA degradosome by studying the dynamics of some of its proposed components at a single molecule level. While RNase Y has been shown to form membrane-associated foci under fluorescence microscopy experiments, other components of the putative RNA degradosome were homogeneously present within the cytosol, as investigated by epifluorescence microscopy (11). These experiments suggest that the RNA degradosome may be dominated by transient interactions, which we wished to quantify by single molecule analyses.

For this task, we employed a highly evolved version of software SMTracker. The new version 2.0 now includes several distinct methods for obtaining diffusion coefficients, to automatically quantify molecule numbers in individual cells, to visualize populations of molecules with different diffusion coefficients within a standardized cell, and analyses of Brownian versus anomalous diffusion. SMTracker 2.0 contains several statistical tools and algorithms to find out if results are significant and are not dominated by overfitting. Importantly, SMTracker 2.0 is now compatible with any computer system and no longer requires additional programs to be operated. We will first introduce the new version 2.0, and then describe investigations of the RNA degradosome, which we interpret to show that together with enolase, RNase Y forms the central hub of the complex, which is highly dynamic and contains peripherally associated proteins such as PfkA and RNase J1. These appear to play minor roles as their dynamics are hardly affected by an inhibition of RNA synthesis, but whose absence affects single molecule dynamics of RNase Y.

## MATERIALS AND METHODS

### Software

SMTracker is a graphical user interface (GUI), custom coded by our lab, that can be easily used by non-mathematicians or non-physicists. Previous versions have been used in several publications (14–18). In this work, we have used the most versatile version of SMTracker, version 2.0, and all the dynamic and statistical analyses and graphs in this paper were generated exclusively by the software. For further information about the techniques applied, please also visit the Supplementary documentation. For specific details of the software and the program for download can be found at https://sourceforge.net/projects/singlemoleculetracker/ where also a User Manual and sample data can be found and explored.

### Construction of strains

Strains of bacteria used in this study were cultivated in LB medium and on solid agar plates at 30°C. Chloramphenicol 5 μg/ml, ampicillin 100 μg/ml, kanamycin 30 μg/ml, spectomycin 100 μg/ml and rifampicin 25 μg/ml were also added where necessary. For SMT experiments, minimal medium S_750_ was used (100 ml: 10 ml 10 × S_750_ salt solution [for 1 l, pH 7.0: 104.7 g of MOPS, 13.2 g of (NH_4_)_2_SO_4_, 6.8 g of KH_2_PO_4_, 12 g of KOH], 1 ml 100 × metal solution [100 ml ddH_2_O: 20 ml of MgCl_2_ (1 M), 7 ml of CaCl_2_ (1 M), 0.5 ml of MnCl_2_ (1 M), 1 ml of ZnCl_2_ (0.1 M), 1 ml of FeCl_3_ (50 mM), 5 ml of Thiamine hydrochloride (2 mg /ml), 17 μl of HCl (2 M)], 2 ml 50% fructose (w/v), 1 ml of 10% l-glutamate (w/v), 40 μl of 1% casamino acids (w/v)) (19). For *Bacillus subtilis* BG214 it was necessary to add methionine (50 μg/ml) and tryptophane (50 μg/ml). The strain in which enolase-mV was induced at low level was treated with 0.01% xylose for 1 h.

All proteins were visualized as mVenus (mV) fusions. *B. subtilis* mV-fusions of RNase Y, RNase J1, RNase J2 and PfkA were created with the use of plasmid pSG1164-mV, generating a C-terminal fusion at the original locus of the gene by a single-crossover event (20). At least 500 bp of the 3'end of the desired gene (excluding the stop codon) were amplified by PCR cloned by Gibson Assembly (21) into the vector in frame with the linker and mV sequence. The necessary Gibson primers had an overhang of at least 20 bp (Supplementary Table S1). For enolase we generated an ectopic fusion in order to express a low level of the enzyme that itself is present in the cell with a high copy number, to be able to rapidly reach single molecule level during SMT. For this purpose, pSG1193-mV was used. Full length *eno* gene (1293 bp) was cloned into the vector in frame with the linker and *mVenus*. Expression of mRNA for the protein was mediated by the xylose promoter. The cloning of all vectors was done in DH5α *Escherichia coli* cells. Deletion strains (*B. subtilis* 168 *ΔrnjB::kan trpC2*, *ΔpfkA::kan trpC2*) were obtained from the Bacillus Genetic Stock Center (Columbus, Ohio) (22). Competent cells of strain RNase Y-mV were transformed with chromosomal DNA from deletion strains. Chromosomal DNA was obtained using innuPREP Bacteria DNA Kit (Analytik-Jena). Bacterial strains and plasmids used in this study are listed in Supplementary Table S2, primers in Supplementary Table S1.

### Western blot

Strains were cultivated in LB medium (30°C) with the addition of the specific antibiotic until mid-exponential growth phase. The enolase-mV fusion was induced with 0.01% xylose for 1 h. Pelleted cells were resuspended in buffer (100 mM NaCl, 50 mM EDTA, 2.5 mg/ml Lysozyme, 0.1 mg/ml RNase, 0.01 mg/ml DNAse). Samples were incubated at 37°C with SDS loading buffer, but were not boiled. Detection was performed with a primary antibody αGFP, polyclonal (1: 500, for 5 h) (23) and secondary antibody goat-anti-Rabbit-IgG, peroxidase-conjugated (1 : 10 000, for 1 h) (Sigma-Aldrich). Protein marker Thermo Scientific™ PageRuler Prestained Protein Ladder was used.

### Fluorescence microscopy and Single-molecule tracking

Epi-fluorescence microscopy was performed in S_750_ minimal medium at 25°C, using a Nikon Eclipse Ti-E, Nikon Instruments Inc with a CFI Apochromat objective (TIRF 100× oil, NA 1.49) and an EMCCD camera (ImagEM X2 EM-CCD, Hamamatsu Photonics KK). Data were evaluated using ImageJ (24).

For SMT, slim-field illumination was obtained using the central part of an expanded beam of a 514 nm laser diode (max. power 100 mW, TOPTICA Beam Smart) was focussed on the back focal plane of the objective. Up to 25% of the intensity was used via a YFP filter set (BrightLine 500/24, Beamsplitter 520 and BrightLine 542/27), generating a laser power density of about 160 W cm^–2^ in the object plane. We have previously shown that cells continue to grow following exposure to this intensity, following the 2 min-long experiments (15). NIS-elements was used for image acquisition. Videos were captured for 3000 frames, with integration time of 40 ms in the case of enolase-mV, RNase Y-mV, *ΔrnjB* RNaseY-mV and *ΔpfkA* RNaseY-mV, or 30 ms for PfkA-mV, RNase J1-mV and RNase J2-mV. The further processing was done with Oufti (25) to set the cell-meshes. Track generation was performed with a minimum track length of five steps u-track (26). The reaching of a single molecule level was tested by analysing bleaching curves in ImageJ. Analytical evaluation was carried out using the SMTracker 2.0, based on an earlier version (27).

### Cross-validation approach on the calculation of the diffusion coefficients

Measurement errors on Diffusion coefficients and fraction sizes have been performed using a cross-validation approach, where the complete dataset has been split into two parts, train and test, in a proportion of 70%/30%. Given the size of our dataset, made more sense to divide the train set into 10 folds randomly and perform the fit on each fold, and showing as a final result the average, ± error, that comes from the standard error of the mean + the 95% confidence interval obtained from the fit procedure.

## RESULTS

### SMTracker 2.0 includes several new analytic tools that are highly useful for the analyses of single molecule dynamics

**Figure 1.**
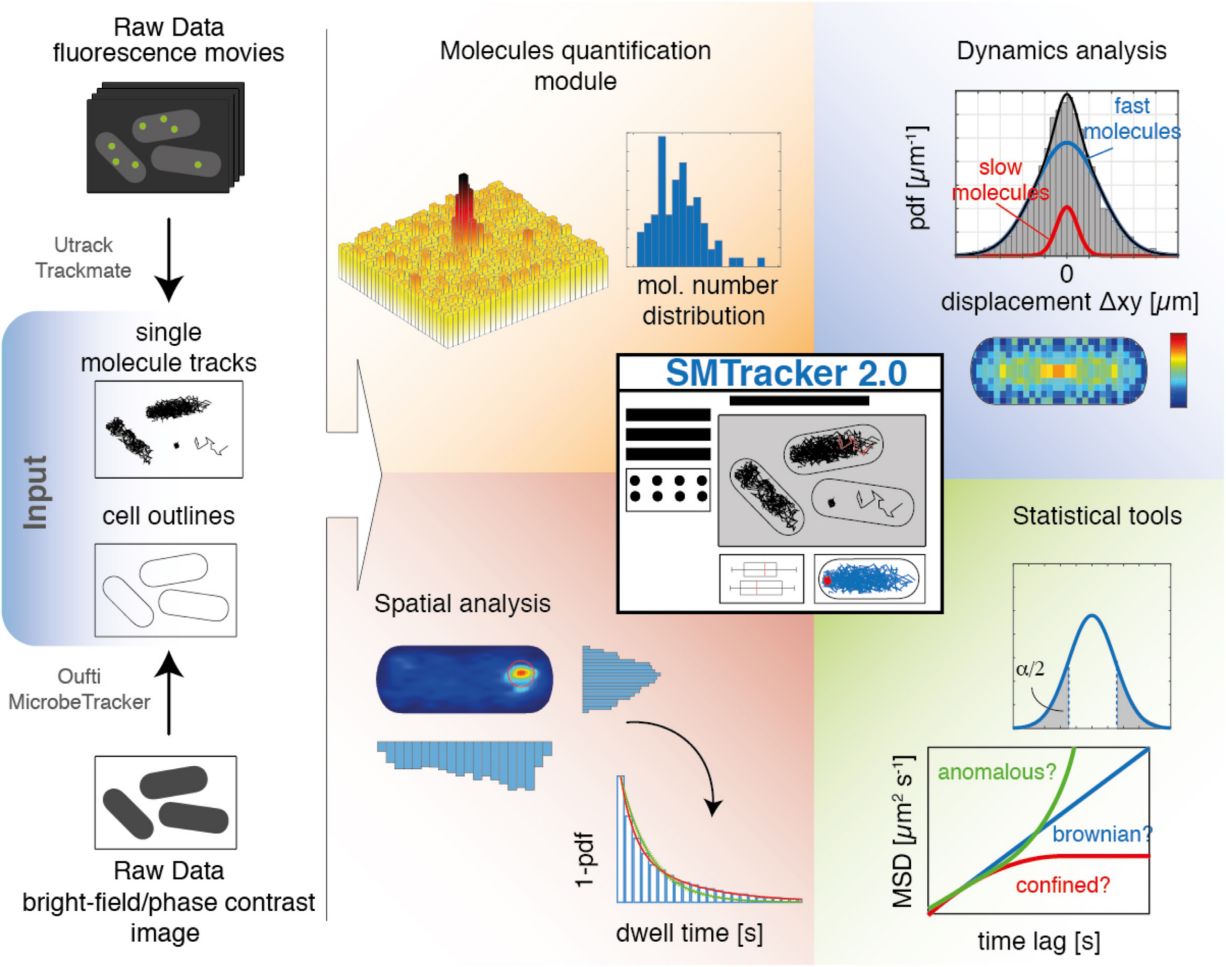
Graphical abstract of SMTracker 2.0 suite.

SMTracker 2.0—built on its previous version 1.0 (27)—was completely revised and extended for this (and future) work. Now it is available as stand-alone version that can run independently of MATLAB. SMTracker 2.0 loads movies acquired from fluorescence microscopy and cell outlines obtained from bright field or phase contrast microscopy (Figure 1). Data analysis is run on a graphical user interphase (GUI) containing seven principle sections dealing with spatiotemporal analyses of single molecule/particle data (Figure 1). In addition to updates on the ensemble-averaged mean squared displacement (MSD) setup, Gaussian mixture modelling (GMM) and squared displacement analyses (SQD) have been improved to detect up to thee different Brownian-diffusive sub-populations. SMTracker 2.0 also contains apparent diffusion (AppD) analyses to determine diffusion constants using each track and time-averaged MSD. With the new tool ‘Clustering’, time-averaged MSD (TAMSD) data are used to classify trajectories using a machine-learning approach by an unsupervised *K*-means clustering algorithm. Spatial localization (SLA) analysis, which comprehends dwelling and confinement events determination, have been completely reviewed and upgraded optimizing both the algorithm and the data visualization.

Three new-fangled modules have been developed for a better understanding of the dynamic behaviour of proteins inside an organism, or *in vitro*. ‘Distance tool’ is a user-driven module that provides information about the trend and the position of a protein with respect to defined points in the cell. A ‘Molecule quantification’ tool takes advantage of the microscopy movies and the pattern of fluorescence emission that is inherent to the fluorescent protein (FP) to estimate the molecule copy number within a certain strain, and even within individual cells. Finally, the ‘Binned speed maps’ tool has been devised that determines areas with varied protein diffusion coefficients within the cell. The latter tool is especially helpful in visualizing diffusive subpopulations in the cell, generating a 2D population diffusion map.

Please note that SMTracker 2.0 requires a certain arrangement of data within folders, explained in the manual. We have also created direct links to a program calculating dwell times in a way different from SMTracker, called ‘SMM Track’ (28), and a link to the useful tool vbSPT from the Elf laboratory, where changes of molecules between different diffusive populations can be predicted (29). Instead of going through lengthy descriptions of SMTracker 2.0, we present experiments on the dynamics of the putative RNA degradosome in *B. subtilis* cells, highlighting most powerful improvements in and additions to the program.

### RNase Y and enolase, but not RNase J1, J2 and PfkA, show changes in protein dynamics in response to inhibition of transcription

We generated a fusion of mVenus to the last coding 500 bp of the *rny* gene; the plasmid was integrated into the *rny* gene locus via single crossover integration, generating a C-terminal RNase Y-mVenus fusion. This way, RNase Y-mVenus was the sole source of the protein expressed in the cell, under control of the original promotor, and expression of the downstream gene *ymdB* was ensured via addition of xylose driving the *pxyl* promoter present within the plasmid. Cells expressing this construct grew with a doubling time indistinguishable from wild type cells, suggesting that the fusion complemented the loss of the wild type protein, because an *rny* deletion leads to a severe growth defect (5,30). In an analogous manner, we generated mVenus fusions to enolase, RNase J1, RNase J2, PNPase, CshA and phosphofructokinase I (PfkA). All strains grew with wild type appearance and doubling times, suggesting that all fusions could functionally replace wild type copies. Using epifluorescence microscopy, RNase Y-mVenus showed a spotty pattern of localization, and likewise RNase J1 and RNase J2, in contrast to PfkA and enolase which revealed a more homogeneous fluorescence within the cytosol (Supplementary Figure S1A). Western blot analyses showed that all fusion were expressed as full length proteins (Supplementary Figure S1B). Please note that the size of RNase J2-mV (83.57 kDa) and PfkA-mV (61 kDa) differs from the literature. The different running behaviour may be due to sample handling, as we avoided boiling of samples. The expression of free mVenus (26.9 kDa) that might occur due to proteolysis was not observed.

For single molecule tracking, we used a system in which the central part of a widened 514 nm laser is projected onto the back focal plane of the objective, generating a small homogeneous illumination at the center of the visible field (sometimes termed ‘slim-field’ microscopy). Images were acquired with an EM-CCD camera at 20 (RNase J1, RNase J2, PfkA, PNPase and CshA and 40 ms (RNase Y and enolase) stream acquisition rate, such that movement of single point spread functions could be captured. Tracks were automatically recorded using u-track program. At the beginning of illumination, all molecules in the cell fluoresce, and after few hundred frames, most molecules are bleached, leaving one or very few molecules per cell that can be tracked. Bleaching curves are evaluated in ImageJ to ensure that single molecule conditions applied at the beginning of the movies that were used for analyses. Because enolase is expressed at high copy number in *B. subtilis* cells, such that single molecule level was only obtained after several thousands of frames, we imaged a strain in which enolase-mVenus is expressed from a gene fusion at the ectopic *amyE* locus, using very low induction of the xylose promoter driving transcription. In this way, only very few molecules of enolase-mVenus are expressed together with many copies of the wild type protein, allowing single molecule level to be rapidly reached.

**Figure 2.**
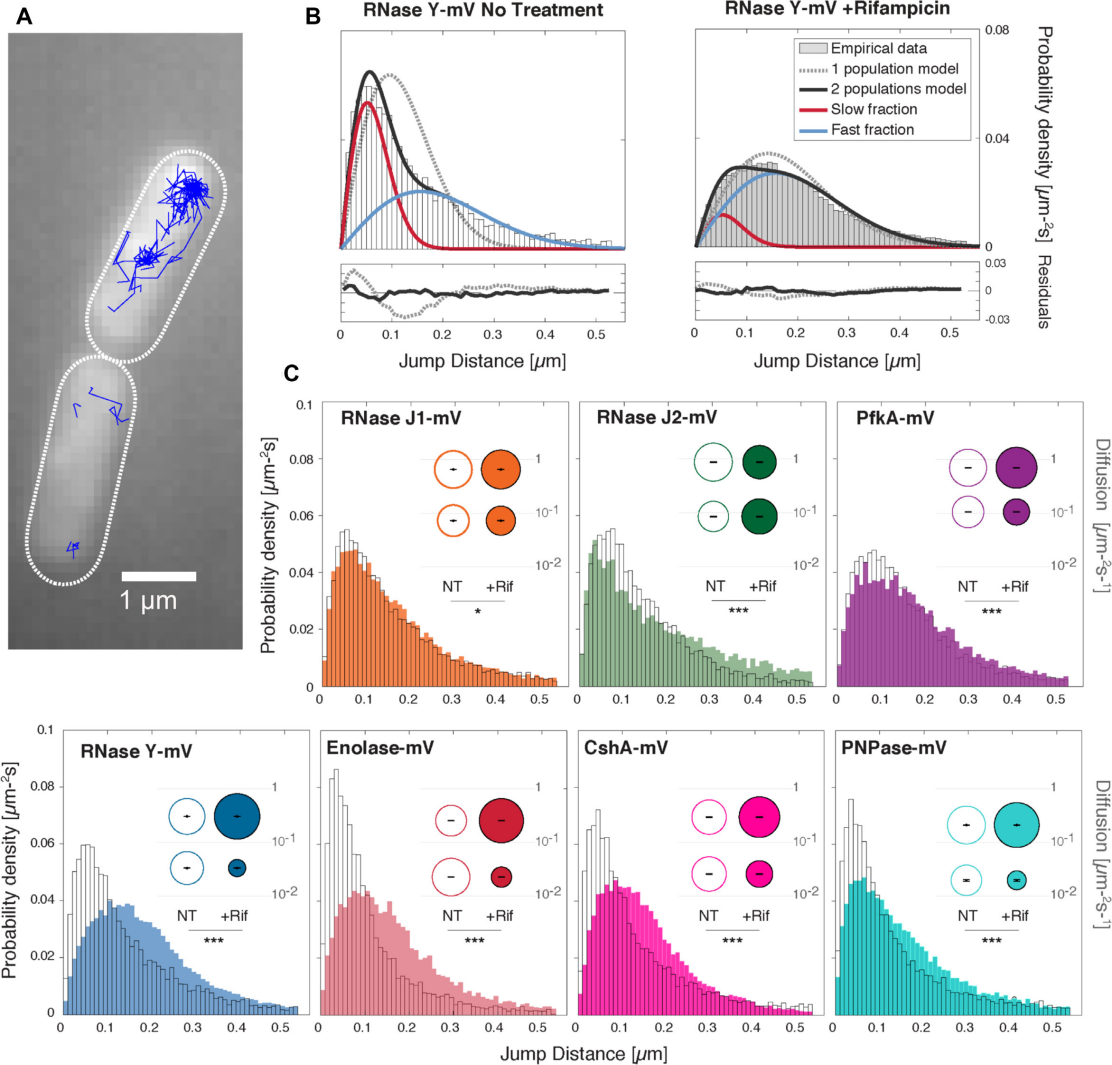
Acquisition and Diffusion analysis pipeline via SMTracker 2.0. (**A**) Examples of trajectories of RNase Y-mVenus. (**B**) Jump distance histograms overlaid with a 2-population model fit for RNase Y-mVenus before (left) and after Rifampicin (Rif) treatment (right). Fitting was performed by a non-linear least squares algorithm. Residuals are shown in the lower panels. (**C**) Jump distance histograms for the 4 RNases, RNA helicase and the two glycolytic enzymes before (no fill) and after Rif treatment (filled with solid color). Inlet: Bubble plots, the size of the bubbles is proportional to that of each diffusive subgroup, and short segments in black indicates the ± confidence interval at 95% of diffusion coefficients. Stars correspond to the result of a Kolmogorov-Smirnov 2-test to determine significant differences between the two distributions. As usual, ***, **, * and ns when the *P*-values are lower than 0.001, 0.05, 0.01 and larger than 0.01, respectively.

Molecule steps and trajectories were analysed using SMTracker 2.0. Figure 2A shows an example of cells with recorded tracks. Only tracks of 5 steps or longer were used for analyses in order to avoid artefacts from very short events. Figure 2B shows the jump distance distribution (as a probability density function) of RNase Y-mVenus, as determined from squared displacement analyses. The grey dotted line shows a single fit to the data, assuming one population of molecules that have a common diffusion coefficient. This single fit cannot explain the observed data, and the ‘residuals’ panels (Supplementary Figure S2) indicate a great deviation of the fit (green line) from the measured values, which are represented by the horizontal line. Red and blue curves are Rayleigh fits assuming two or three populations, respectively, one with a low and one with a higher diffusion constant in case of two populations. Taken together (black solid line), two assumed populations can explain the observed distribution of steps rather well, which can also be seen in the corresponding residuals panel (Supplementary Figure S2). Likely, the fast-mobile fraction corresponds to freely diffusive RNase Y molecules, while the slow/static fraction is presumably composed of RNase Y molecules bound to mRNA and/or the putative degradosome. In fact, the diffusion constant determined for the static fraction with *D* = 0.031 ± 0.001 μm^2^/s (Table 1) is similar to that determined for translating 70S ribosomes/polysomes with *D* = 0.055 μm^2^/s (31), suggesting that this fraction of RNase Y is within a large protein/RNA complex. The diffusion constant of the fast-mobile fraction with *D* = 0.3 ± 0.002 μm^2^/s is quite low for a freely diffusive protein, compared with diffusion coefficients obtained before (18,28) (free mVenus has a coefficient of 5 μm^2^/s within *B. subtilis* cells (32)). Possibly, the mobile fraction of RNase Y is composed of freely diffusing dimers (see further below for this point) as well as of molecules in transition towards binding to mRNA. Also, tracking of RNase Y at 40 ms integration time may miss out on some very rapidly diffusing molecules, leading to an underestimation of *D*. We will pick up this point further below.

**Table 1.** Diffusion coefficients and fraction sizes

	RNase Y	RNase Y with Rif
*pop*1 (%)	48.2 ± 0.6	9.84 ± 0.6
*pop*2 (%)	51.8 ± 0.3	90.2 ± 0.3
*D*1 (μm^2^ s^−1^)	0.031 ± 0.0008	
*D*2 (μm^2^ s^−1^)	0.3 ± 0.002	
	RNase J1	RNase J1 with Rif
*pop*1 (%)	38.5 ± 0.8	35.3 ± 0.8
*pop*2 (%)	61.5 ± 0.7	64.7 ± 0.7
*D*1 (μm^2^ s^−1^)	0.072 ± 0.002	
*D*2 (μm^2^ s^−1^)	0.69 ± 0.01	
	RNase J2	RNase J2 with Rif
*pop*1 (%)	37 ± 2	51.2 ± 2
*pop*2 (%)	63 ± 2	48.8 ± 2
*D*1 (μm^2^ s^−1^)	0.084 ± 0.004	
*D*2 (μm^2^ s^−1^)	0.85 ± 0.03	
	Enolase	Enolase with Rif
*pop*1 (%)	59.3 ± 1	14.5 ± 1
*pop*2 (%)	40.7 ± 0.7	85.5 ± 0.7
*D*1 (μm^2^ s^−1^)	0.022 ± 0.0007	
*D*2 (μm^2^ s^−1^)	0.26 ± 0.004	
	PfkA	PfkA with Rif
*pop*1 (%)	38.5 ± 1	28.1 ± 1
*pop*2 (%)	61.5 ± 1	71.9 ± 1
*D*1 (μm^2^ s^−1^)	0.087 ± 0.004	
*D*2 (μm^2^ s^−1^)	0.67 ± 0.01	
	CshA	CshA with Rif
*pop*1 (%)	39.3 ± 0.7	15.1 ± 0.7
*pop*2 (%)	60.7 ± 0.6	84.9 ± 0.6
*D*1 (μm^2^ s^−1^)	0.02 ± 0.0008	
*D*2 (μm^2^ s^−1^)	0.21 ± 0.003	
	PNPase	PNPase with Rif
*pop*1 (%)	50 ± 0.7	30.1 ± 0.7
*pop*2 (%)	50 ± 0.6	69.9 ± 0.6
*D*1 (μm^2^ s^−1^)	0.026 ± 0.0006	
*D*2 (μm^2^ s^−1^)	0.3 ± 0.004	

ϵ in this context means <10^–6^. The low confident interval amplitude is due to the high number of data.

According to tracks obtained for RNase Y, 48.2 ± 0.6% of the molecules were in a slow-mobile state, and thus likely actively involved in mRNA degradation or RNA processing, while 51.8 ± 0.3% are in the mobile state (Table 1), illustrated by the size of the bubbles in Figure 2C, blue panel. We further analysed RNase Y and the other four proteins using two population fits, which also explained data much better for the other RNases and glycolytic enzymes than single fits (Supplementary Figure S2). Thus, for RNase J1, RNase J2, PNPase, CshA, PfkA and enolase, we found a slow mobile/static fraction similar to that found for RNase Y (Figure 2C, Table 1). The fact that these slow diffusion constants varied by a factor of more than two may indicate that e.g. RNase J1 or enolase may be present in additional complexes, because at the fast acquisition rates used, we cannot distinguish between different slow-mobile states of molecules. The fact that enolase, PfkA and RNase J molecules had different values for D_2_ can be explained by their different sizes, although the *D* of 0.26 ± 0.004 μm^2^/s is very low for this 46 kDa protein. At present, we have no explanation for this unusually low mobility for enolase, with about 60% of molecules even being in the slow mobile/static state (Table 1).

Importantly, Figure 2B shows that for RNase Y, there is a strong shift of step sizes towards larger steps after addition of rifampicin, which lowers transcription levels by blocking RNA polymerase (RNAP) activity. Note that the concentration of 25 μg/ml we used is more than 400 fold above the MIC (33), but less than a maximum of 100 μg/ml employed in a study to completely block RNA synthesis (34), because at the latter concentration we observed cell death 30 min after addition of rifampicin, as judged from cells becoming extremely fluorescent, during SMT acquisition. We did not observe this effect with 25 μg/ml, so we chose this lower concentration, where transcription may not be completely blocked, but where cells are alive, in order to avoid artefacts from dying cells in which molecules may move very differently than under physiological conditions.

The low-mobility fraction (red) strongly decreased in favour of the fast-mobile fraction (blue). The fraction of slow-mobile RNase Y molecules decreased from about 48 to 9.84 ± 0.6% (Table 1), while the fraction of mobile molecules increased concomitantly (Figure 2C, blue panel). Thus, loss of substrate allows slow mobile RNase Y molecules to become mobile.

We also treated cells expressing the other putative RNA degradosome components with rifampicin to test if mobilities change similar to what was observed for RNase Y. Note that to better compare fraction sizes between conditions we kept *D* values for treated cells similar to those of non-treated cells, although when analysed separately, *D* values moderately differ in addition to fraction sizes, between the two conditions. The size of the fractions found in untreated or treated cells is represented as bubbles in panels C. A strong shift in RNase Y mobility as well as of that of PNPase, CshA and enolase can easily be seen in Figure 2C by the overlays of tracks from exponential growth (not filled) and those from rifampicin-treated cells (filled bars), but not to a considerable extent for RNase J1 and J2, or for PfkA. In contrast to RNase Y, RNase J2 molecules became even more static, the population increased from 37 ± 2% to 51.2 ± 2% in response to inhibition of transcription (Table 1). Thus, RNase Y becomes mobile due to a shortage in RNA substrates to bind to, as expected, and for enolase, apparently the same holds true, suggesting that many enolase molecules interact with mRNA (likely via RNase Y) as a component of the RNA degradosome. Conversely, our findings suggest that RNase J1, RNase J2 and PfkA molecules are not quantitatively part of the degradosome, as compared to RNase Y.

### RNase Y, PNPase, CshA and enolase change their pattern of localization in response to inhibition of transcription

We wished to obtain spatial information on changes occurring to putative RNA degradosome components in response to a reduction in cellular RNA levels. We therefore projected tracks obtained into a standardized cell of 1 × 3 μm size, an average size for growing *B. subtilis* cells. We sorted tracks into those that show an extended period of little to no movement, termed ‘confinement’, where molecules stop much longer than would be expected from stochastic slow-mobile events during free diffusion. Confined movement occurs when molecules become bound to larger structures; for RNase Y molecules, this would most likely correspond to mRNA binding, or binding to components of the putative degradosome. We chose 8 steps or more as indicative of confined motion, and a radius of 106 nm, corresponding to the pixel size of the camera, or about 2.5 times the localization error.

**Figure 3.**
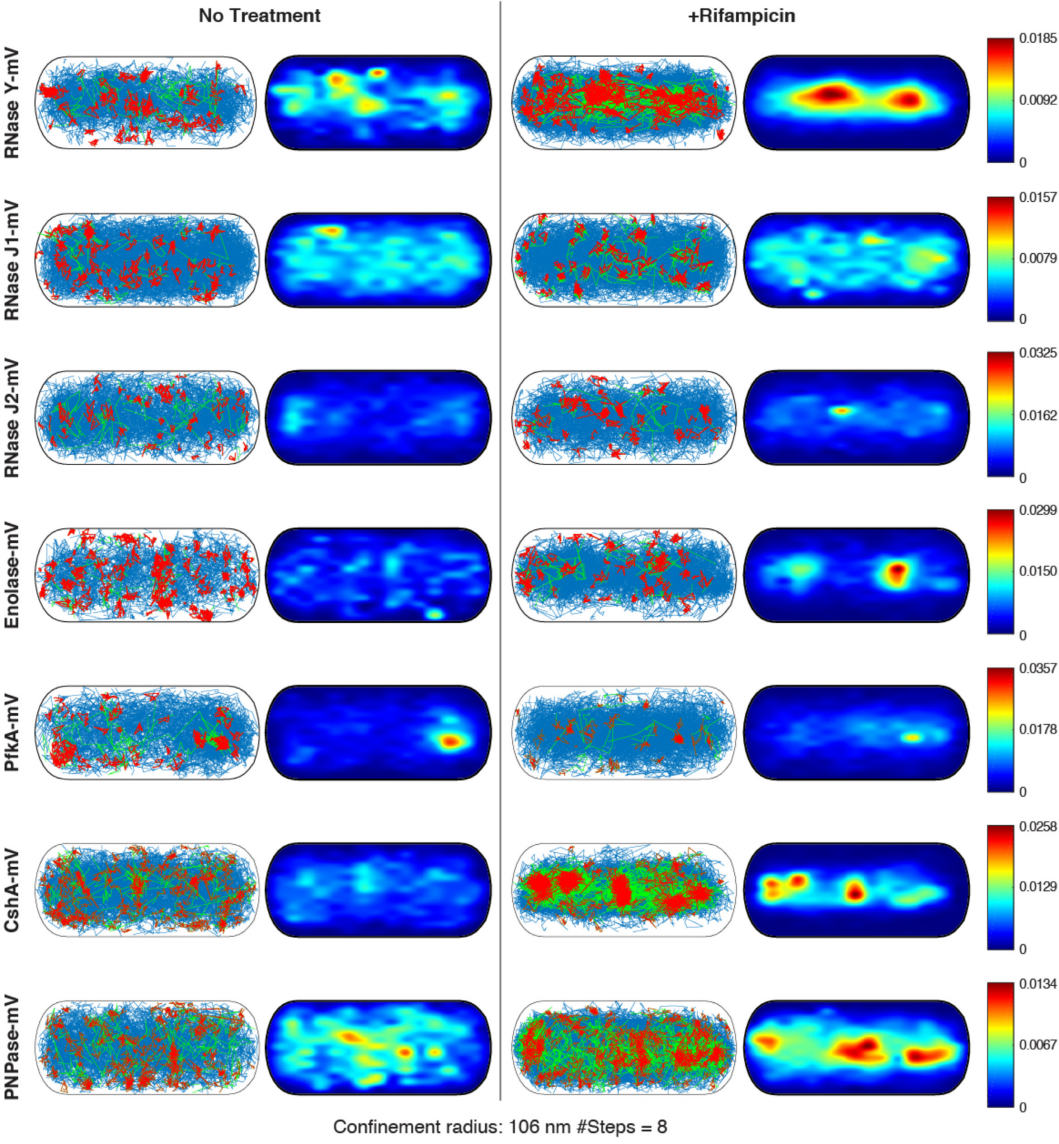
Confinement maps and heat maps. Confined tracks are shown in red, tracks showing no confined motion in blue, green tracks contain confined as well as non-confined motion. Colour codes on the right indicate low (‘0’ blue, to high, red, likeliness of presence). Cells have a width of 1 μm and a length of 3 μm, roughly the average of exponentially growing cells.

Figure 3 shows that RNase Y moves throughout the cell (blue tracks), and shows confined motion mostly towards the periphery of the cell, i.e. close to the cell membrane. In the heat map, where localization probability increases from blue to red, RNase Y also shows a tendency towards the membrane. This pattern changes dramatically after addition of rifampicin. Under this condition, RNase Y relocates from the periphery to the cell center. This trend can also be seen for confined tracks, which are much more accumulated within the cell, away from the membrane, than during exponential growth. Thus, depletion of (m)RNA strongly affects the pattern of localization of RNase Y, and its preferred sites of confined motion, i.e. it is likely places of activity.

A comparable effect of relocation was observed for enolase, where rifampicin addition was accompanied by an accumulation towards the cell center (Figure 3). This effect was visually less pronounced than for RNase Y, but still noticeable by eye; confined tracks of enolase also shifted away from the periphery towards the cell middle. An effect similarly striking as for RNase Y was seen for PNPase and for CshA (Figure 3). However, neither RNase J1, nor J2, nor PfkA revealed considerable differences in the confinement maps or the heat maps between unperturbed growth and inhibition of transcription. RNase J1 showed a weak accumulation towards the membrane in the heat map, which appeared to be weakened after treatment, while RNase J2 showed a weak accumulation after treatment in the cell center, which was not apparent during exponential growth. Thus, RNase Y, CshA, PNPase and enolase show similar responses to an arrest in transcription in terms of single molecule dynamics (Figure 2) and spatial redistribution (Figure 3), while the other proposed members of the degradosome show only mild to no changes.

**Figure 4.**
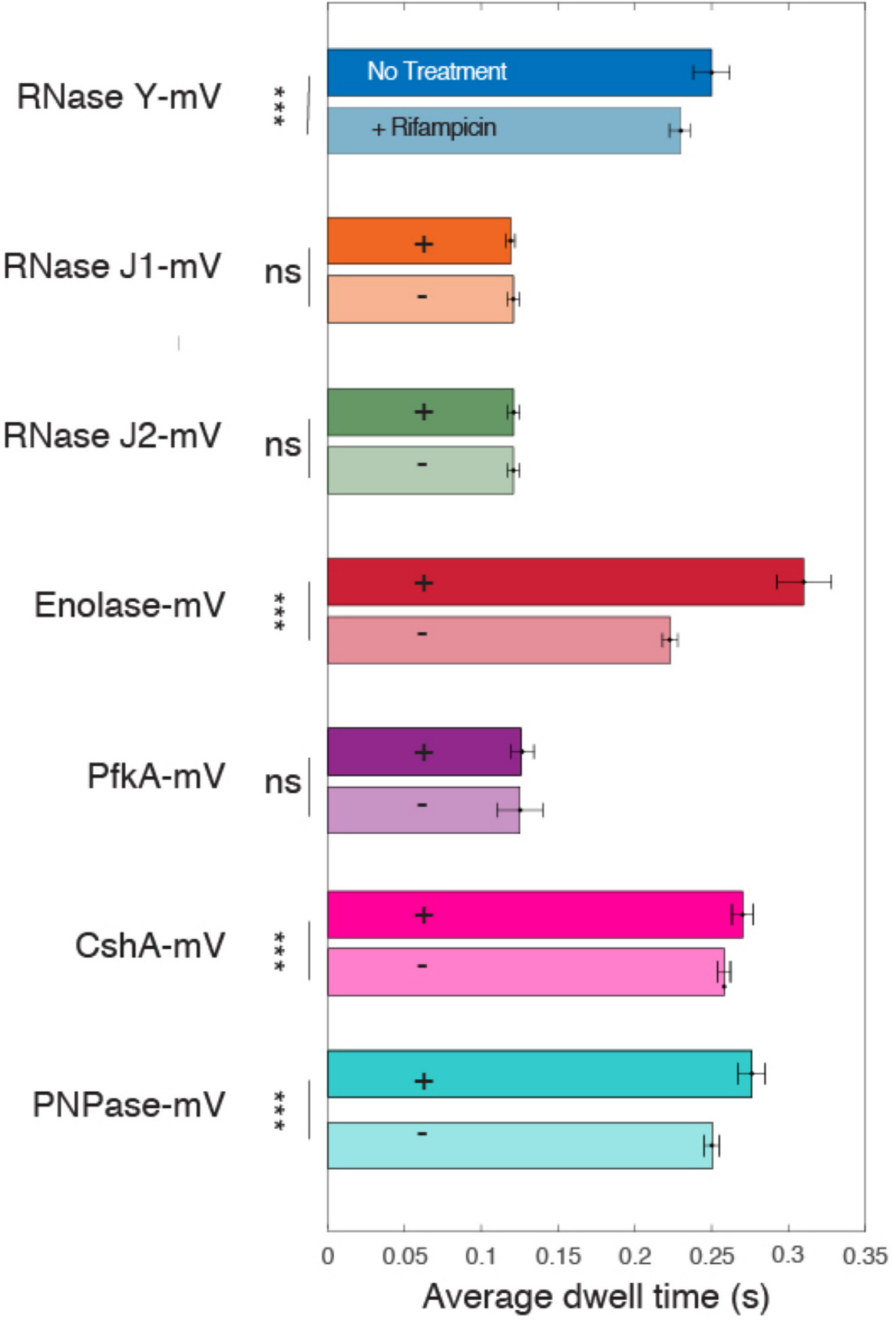
Dwell times for enzymes under investigation. As usual, *** indicates highly significant changes (*P*-value < 0.001), n.s. statistically not significant (*P*-value > 0.1). Note that while actual values for dwell times will be higher *in vivo* due to photobleaching effects, differences between individual proteins/conditions can be accurately determined.

We also determined average dwell times for molecules. Here, the time spent within a radius that can be manually defined, or is determined to be three times the localization error (determined from the intercept of the MSD curve with the y-axis) is scored for x intervals. Representative curves showing dwell time determinations can be seen in Supplementary Figure S3. Exponential decay curves are fitted to the data, and an average dwell time ‘tau’ is determined for all molecules, and in case two decay curves can better explain the obtained dwell time probabilities, tau 1 and tau 2 are calculated, together with corresponding sizes of populations, which one can interpret as molecules resting stochastically (tau 1), and molecules arresting because of e.g. binding events, which are much longer than stochastic ‘no-displacement’ events (Table 3). Figure 4 illustrates that average dwell times for RNase Y decreased significantly following rifampicin treatment, and even more pronounced for enolase, while PNPase and CshA showed less pronounced, yet significant reductions in dwell times. This was not observed for RNase J1 and J2, nor for PfkA, which did not reveal significant changes in response to lowering of mRNA levels. Of note, while our analyses contain many long tracks that allow for a sufficiently reliable deduction of dwell times, actual dwell times *in vivo* will be longer, as our analyses involve bleaching of molecules, which leads to an underestimation of dwell times. Additionally, lack of changes may not mean absence of interaction with e.g. mRNA in this case: it has been shown that RNase J1 is responsible for the degradation of a considerable fraction of *B. subtilis* mRNAs (35). Possibly, RNase J1 molecules undergo non-productive interactions with different, weakly binding proteins in the absence of high affinity binding substrates. Note that dwell times cannot be directly compared between protein fusions, because RNase Y and enolase were tracked using 40 ms integration time, and the other fusions with 20 ms.

### SMTracker 2.0 provides a tool for automated counting of molecules in individual cells

**Figure 5.**
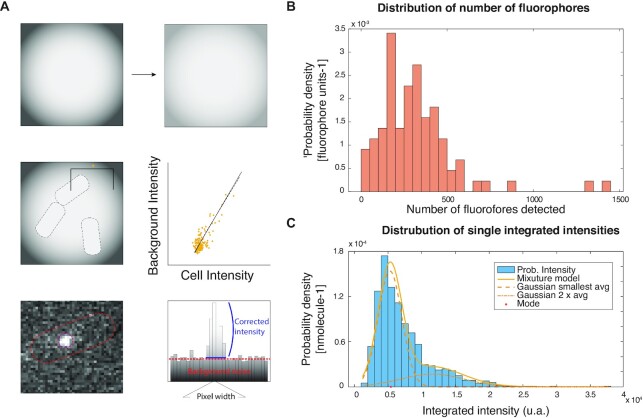
Automated fluorescence-based quantification of RNase Y molecule copy number. (**A**) Scheme of the procedure to quantify the number of fluorophores in the movies, where first a correction of the irregular illumination is made (first row), then an estimation of the autofluorescence using the relationship between cells lacking any fluorescent protein fusion and background (second row) and finally, estimation of the fluorescent intensity of the spots using as center the detected position of the protein in the track detection algorithm inside a circle of radius 3 pixels. (**B**) Distribution density function of the number of detected fluorophores in all cells. (**C**) Distribution density function of 2004 integrated spot intensities. In the best estimation, there are two populations of average integrated intensity I1 ∼ = 5284 and I2 ∼ = 11326 u.a., with a proportion of 68%/32%, suggesting dimerization of RNase Y. Estimation of number of fluorophores after accounting for dimer formation and simulation corrections = 485.

We implemented a novel tool to obtain information on average molecule numbers, as well as numbers of molecules in individual cells. The approach calculates average signal intensity of single fluorophores and calculates molecule numbers from fluorescence intensity captured in the first frames. In order to account for background fluorescence, it is important to subtract fluorescence found in cells not expressing any fluorophore/fluorescent protein fusion. Figure 5 illustrates our approach: from samples lacking any cells, uneven illumination is captured, and background fluorescence is scored from cells not expressing any fluorescent protein fusion. Having these values, initial fluorescence intensity of cells carrying fluorophores/FP fusions is captured, until after bleaching, signals from single molecules can be quantified to yield an average number of intensity per single molecule. As opposed to YFP-tracking/photobleaching based SMT, in live cell PALM tracking, conditions would need to be established to photoactivate all molecules within the first few frames, in order to employ this approach.

For RNase Y, we determined an average molecule number of 485 per cell, and thus a medium copy number, as opposed to low copy numbers determined for e.g. DNA repair protein Ada (36) or cyclic di-GMP synthetases DgcK (14), which are not even present as single copy in all cells of a culture, or high copy numbers of several thousand for e.g. ribosomes or RNA polymerase (37). This number lies in between that obtained in a report using mass spectrometry-based determination of protein copy numbers, of 222 copies in a richer and 716 molecules in a poorer growth medium (38). Assuming a rounded number of 500 molecules per cell, our data suggest that based on our finding of about 46% of statically positioned RNase Y molecules (Figure 2, Table 1), 230 molecules are engaged in RNase activity within the degradosome, while an average number of 270 molecules are diffusing through the cell in search of substrate and binding partners. Of interest, we also observed the existence of spots showing at least two discrete bleaching steps, indicating that a number of RNase Y molecules either form dimers, or that at least two molecules are in close proximity. This finding is in agreement with RNase Y forming dimers *in vitro* (39). Figure 5C shows that 32% of single spots contain two molecules, as the mean of the smaller population contains twice the integrated density of the mean of the larger (monomeric fraction). Because of stochastic bleaching of molecules, these findings do not mean that 68% of RNase Y molecules are monomers, but that clearly, a considerable fraction of molecules are accompanied by additional copies of RNase Y, implying that RNase Y clusters likely contain more than one RNase Y molecule. As a second example, we determined the copy number of enolase (in this case expressed from the original gene locus, as sole copy of the protein), shown to be present between 5000 and 10 000 copies per cell (38), for which we determined an average number of 3900 ± 175 (Supplementary Figure S5). Likely, fluorescence signals will be close to saturation at this high level, wherefore the determined number may be an underestimate.

We benchmarked results obtained from the Molecule quantification tool with modelled data (Supplementary Tables S3 and S4), in order to judge the capabilities of this method. Supplementary Figure S4 shows that for 10 molecules or less, the algorithm performs very well, while from 20 molecules up, a stable underestimation of 15% appears to take place. Thus, for 20 molecules or more, obtained numbers should be adjusted by addition of 15% (this was already done for the RNase Y determination above). Note that also, not all fluorescent proteins may be in an excitable state within the cell (20% estimated for GFP (40), and likely much less for the fast maturing mVenus). Therefore, obtained numbers are also technically an underestimate of actual numbers within the cells.

### Active transcription affects subcellular diffusion of RNase Y

**Figure 6.**
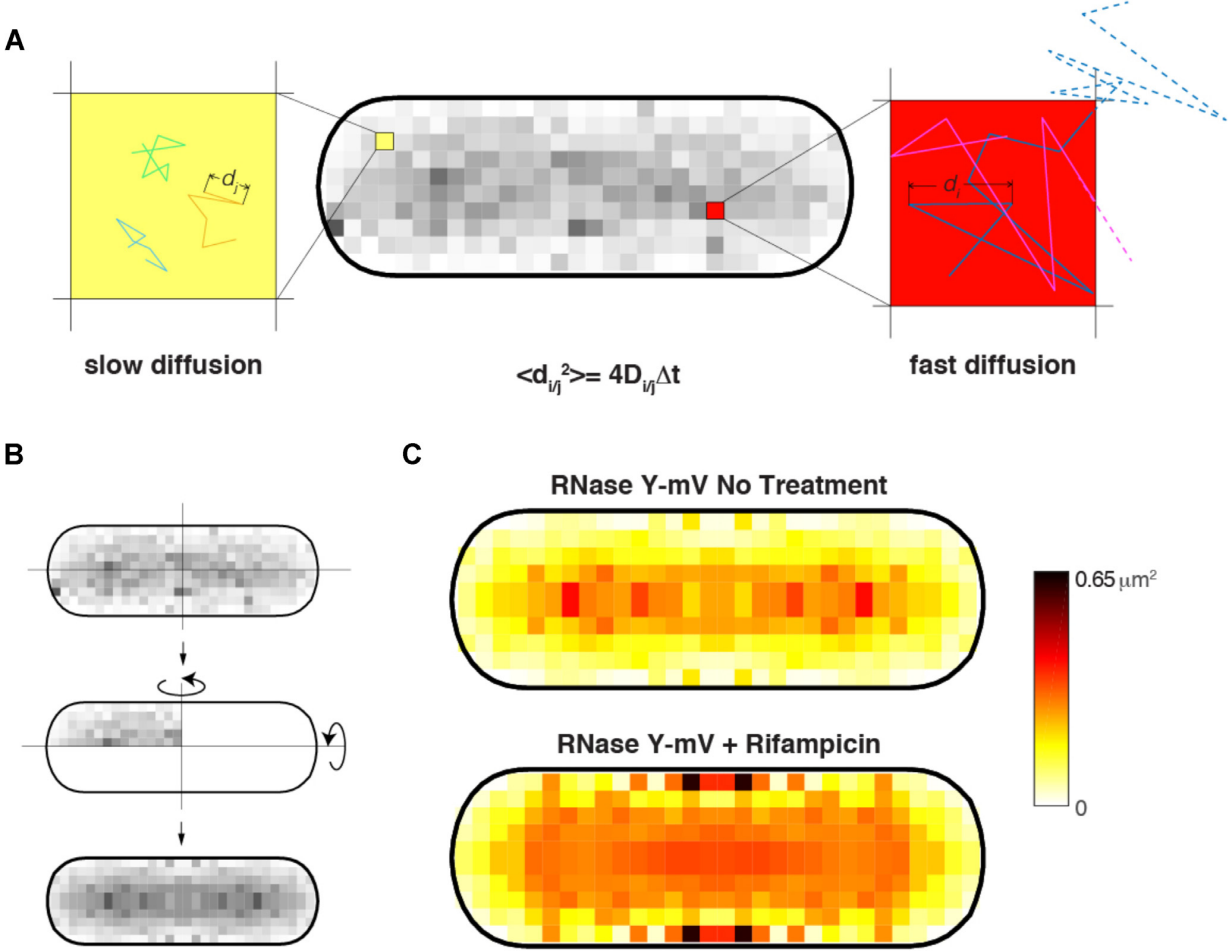
Binned speed maps. (**A**) Average of the apparent diffusion from each two consecutives detections of all trajectories has been calculated and binned in a grid of 100 nm. (**B**) Considering the absence of polarization, the map has been mirrored for a better visualization. (**C**) Binned speed maps applied to RNase Y before and after Rifampicin treatment.

A tool we term ‘Binned speed map’ can visualize average diffusion constants in the cell with 100 nm resolution. Figure 6 reveals that diffusion of RNase Y is much slower close to the cell membrane than within the cell center. While this is likely based on the formation of RNA degradosome complexes containing RNase Y, leading to confined motion, we note that differences in diffusion constants span from close to zero to 0.65 μm^2^/s. This considerable difference is largely lost upon inhibition of transcription (Figure 6C), showing that RNA synthesis strongly affects the subcellular diffusion of RNase Y, and possibly of other proteins. We believe that this tool will be of high relevance for scientists studying subcellular movement of molecules within live cells.

### Absence of PfkA, and to a minor degree of RNase J2, leads to strong differences in RNase Y molecule dynamics

**Figure 7.**
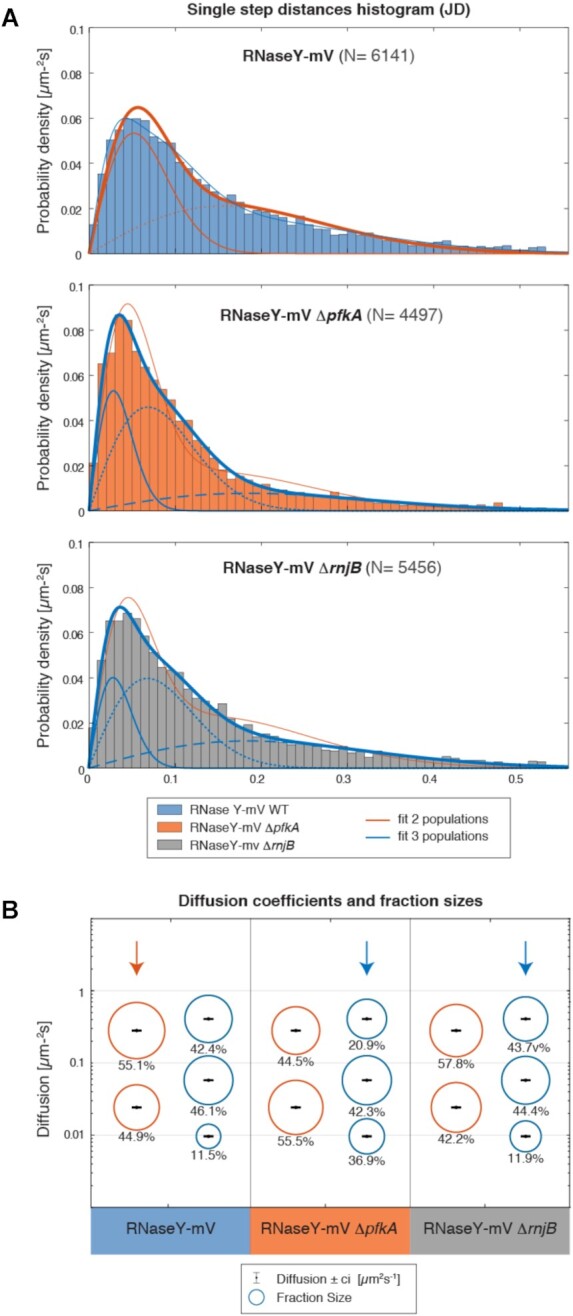
RNase Y-mV dynamics compared between wild type cells and two different mutant backgrounds, deletion of pfkA or of rnjB (encoding for RNase J2). (**A**) Jump Distance histograms of RNase Y-mV in wild type, pfkA or rnjB mutant cells. (**B**) Bubble plots comparing different estimation of the number of populations. For the deletions, three populations seem the best option.

We did not observe strong changes in RNase J1, RNase J2 and PfkA molecules upon inhibition of transcription (Figures 2 and 3). Because this does not necessarily mean that a small number of molecules may be implicated in the formation of the RNA degradosome, we used changes of RNase Y dynamics as an indicator of whether RNase J2 and PfkA may influence RNA degradosome motility by tracking RNase Y-mVenus in the corresponding deletion strains {note that a strain lacking RNase J1 is not viable (35)}. Figure 7A shows that RNase Y single molecule dynamics were markedly altered in the absence of PfkA, and to a minor degree in cells lacking RNase J2. Please note that in order to better compare changes in dynamics, *D* values best-fitting for all three types of cells were determined and kept constant, such that only fraction sizes vary. Thus, in reality, *D* as well as population sizes are different, but in our analyses, we consider changes in molecule dynamics by solely projecting them into changes in fraction sizes. This is a further handy possibility of SMTracker 2.0. For this reason, numbers between Figures 2 and 7 are slightly different. Clearly, in Δ*pfkA* cells, the size of the static/slow mobile of RNase Y molecules increased considerably, from about 40 to 56% (Figure 7B, Table 2). Therefore, RNase Y became considerably more static in the absence of PfkA. A similar but smaller effect was seen in Δ*rnjB* cells (Figure 7A and B). Possibly, RNase Y molecules become less efficient in the absence of PfkA or RNase J2, or can less efficiently change between bound and rebound states.

**Table 2.** Diffusion coefficients and fraction sizes RNase Y upon deletion of *pfkA* or of *rnjB*

	RNase Y	RNase Y Δ*pfkA*	RNase Y Δ*rnjB*
*pop*1 (%)	44.9 ± 1	55.5 ± 1	42.2 ± 1
*pop*2 (%)	55.1 ± 1	44.5 ± 1	57.8 ± 1
*D*1 (μm^2^ s^−1^)	0.025 ± 0.001		
*D*2 (μm^2^ s^−1^)	0.29 ± 0.01		
*pop*1 (%)	11.5 ± 0.7	20.9 ± 0.7	11.9 ± 0.7
*pop*2 (%)	46.1 ± 0.9	42.3 ± 0.9	44.4 ± 0.9
*pop*3 (%)	42.4 ± 5	36.9 ± 5	43.7 ± 5
*D*1 (μm^2^ s^−1^)	0.0081 ± *ϵ*		
*D*2 (μm^2^ s^−1^)	0.16 ± 0.2		
*D*3 (μm^2^ s^−1^)	0.3 ± 0.2		

ϵ in this context means <10^–3^. The low confident interval amplitude is due to the high number of data.

**Table 3.** Dwell time estimation (min four steps)

	No treatment	+ Rifampicin
**RNase Y**		
*Average residence time (s)*	0.25 ± 0.008	0.23 ± 0.005
*τ (1-comp.) (s)*	0.21 ± 0.006	0.19 ± 0.004
*τ_1_ (2-comp.) (s)*	0.18 ± 0.004	0.17 ± 0.002
*Fraction τ_1_ (%)*	82.4 ± 3.78	86.8 ± 2.15
*τ_2_ (2-comp.) (s)*	0.43 ± 0.048	0.43 ± 0.041
*Fraction τ_2_ (%)*	17.6 ± 3.78	13.2 ± 2.15
**RNase J1**		
*Average residence time (s)*	0.119 ± 0.003	0.121 ± 0.004
*τ (1-comp.) (s)*	0.1 ± 0.0025	0.099 ± 0.002
*τ_1_ (2-comp.) (s)*	0.086 ± 0.005	0.089 ± 0.001
*Fraction τ_1_ (%)*	62.7 ± 15	84.4 ± 2.18
*τ_2_ (2-comp.) (s)*	0.14 ± 0.018	0.22 ± 0.017
*Fraction τ_2_ (%)*	37.3 ± 15	15.6 ± 2.18
**RNase J2**		
*Average residence time (s)*	0.121 ± 0.004	0.121 ± 0.004
*τ (1-comp.) (s)*	0.1 ± 0.002	0.1 ± 0.0028
*τ_1_ (2-comp.) (s)*	0.087 ± 0.005	0.087 ± 0.001
*Fraction τ_1_ (%)*	61.4 ± 14.2	82.4 ± 2.8
*τ_2_ (2-comp.) (s)*	0.14 ± 0.02	0.21 ± 0.02
*Fraction τ_2_ (%)*	38.6 ± 14.2	17.6 ± 2.8
**Enolase**		
*Average residence time (s)*	0.310 ± 0.012	0.223 ± 0.005
*τ (1-comp.) (s)*	0.27 ± 0.008	0.19 ± 0.007
*τ_1_ (2-comp.) (s)*	0.19 ± 0.004	0.15 ± 0.006
*Fraction τ_1_ (%)*	56.8 ± 3.09	57.5 ± 8.56
*τ_2_ (2-comp.) (s)*	0.42 ± 0.02	0.26 ± 0.02
*Fraction τ_2_ (%)*	43.2 ± 3.09	42.5 ± 8.56
**PfkA**		
*Average residence time (s)*	0.126 ± 0.005	0.125 ± 0.010
*τ (1-comp.) (s)*	0.11 ± 0.004	0.094 ± 0.002
*τ_1_ (2-comp.) (s)*	0.078 ± 0.004	0.088 ± 0.001
*Fraction τ_1_ (%)*	53.9 ± 7.51	92.8 ± 1.07
*τ_2_ (2-comp.) (s)*	0.15 ± 0.01	0.41 ± 0.05
*Fraction τ_2_ (%)*	46.1 ± 7.51	7.23 ± 1.07%
**CshA**		
*Average residence time (s)*	0.270 ± 0.007 s	0.258 ± 0.004 s
*τ (1-comp.) (s)*	0.23 ± 0.0063 s	0.23 ± 0.0035 s
*τ_1_ (2-comp.) (s)*	0.2 ± 0.0059 s	0.2 ± 0.0056 s
*Fraction τ_1_ (%)*	81.6 ± 5.29%	76.9 ± 7.45%
*τ_2_ (2-comp.) (s)*	0.47 ± 0.071 s	0.36 ± 0.039 s
*Fraction τ_2_ (%)*	18.4 ± 5.29%	23.1 ± 7.45%
**PNPase**		
*Average residence time (s)*	0.276 ± 0.009 s	0.250 ± 0.005 s
*τ (1-comp.) (s)*	0.23 ± 0.005 s	0.22 ± 0.0025 s
*τ_1_ (2-comp.) (s)*	0.2 ± 0.0042 s	0.18 ± 0.0056 s
*Fraction τ_1_ (%)*	83.8 ± 3.53%	58.4 ± 8%
*τ_2_ (2-comp.) (s)*	0.5 ± 0.058 s	0.29 ± 0.016 s
*Fraction τ_2_ (%)*	16.2 ± 3.53%	41.6 ± 8%

Interestingly, in mutant cells, the step size distribution of RNase Y could be better explained assuming three populations (Figure 7A). The triple fit shown in blue describes observed data much better than the double fit shown in red. This finding is very valuable, because it reflects the importance of verifying single molecule tracking data by different types of analyses, and optimally under different conditions or mutant backgrounds. Assuming three populations for RNase Y does not violate the empirical probability density functions (pdfs) of the steps taken by molecules, but may be overfitting of data, while this is not the case for the data obtained from the two mutant strains. Assuming three populations could reflect RNase Y molecules being positioned in the membrane-associated RNA degradosome (static), molecules possibly being associated with the Y-complex (medium-mobile), and molecules freely diffusing along the membrane or through the cell (high mobile). Further experiments are required to reveal if RNase Y may have more than 2 mobility states. This question does not compromise our findings of changes between static/slow molecules after inhibition of transcription or in mutant backgrounds. Considering three populations, RNase Y still becomes less mobile in the absence of PfkA, and to a smaller extent in cells devoid of RNase J2: while medium-mobile fractions remain relatively similar between wild type and mutant cells, fast-mobile fractions decrease in favour of static fractions (Figure 7B, Table 2).

Consistent with these findings, we observed that cells expressing RNase Y-mVenus as sole source of the proteins grow with a doubling time indistinguishable from that of cells lacking the fusion (i.e. expressing wild type RNase Y), but show a growth defect in the absence of PfkA or of RNase J2 (Supplementary Figure S6), while *pfkA* or *rnjB* mutant cells grow with wild type-like doubling time. These data suggest that while the fusion of RNase Y and mVenus can complement the function of wild type RNase Y in wild type cells, absence of phosphofructokinase or of RNase J2 somewhat affects the activity of the fusion protein.

In summary, our data indicate that the absence of Phosphofructokinase has a noticeable effect on the motion of RNase Y, suggesting that PfkA does play a role in the formation of RNA degradosome, and likewise RNase J2, albeit not a pivotal role. We propose that both proteins are only peripherally associated with the degradosome and may optimize RNaseY activity, directly or indirectly.

## DISCUSSION

Single molecule tracking is a highly powerful method. It has greatly increased the resolution scientists can employ to study protein localization, interaction and dynamics in live cells (41). Biochemical experiments have put forward evidence suggesting that RNase Y from *B. subtilis* forms a complex implicated in general mRNA decay at the cell membrane, involving different RNases, an RNA helicase as well as two glycolytic enzymes (39,42,43). Cell biological evidence for this could not be obtained from conventional fluorescence microscopy (11). Here, we provide evidence that PNPase, helicase CshA and enolase change their subcellular dynamics in a way similar to RNase Y upon depletion of mRNA, and also lose membrane-proximal confined motion similar to RNase Y, strongly indicating that many PNPase, CshA and enolase molecules are present within the membrane-associated RNA degradosome complexes. This agrees with a direct interaction of RNase Y and PNPase (44), and with interaction of CshA with RNase Y, PNPase, enolase and phosphofructokinase (42). For phosphofructokinase and RNase J2, we obtained more indirect, nevertheless convincing evidence that they participate in RNase Y-associated activities, as suggested by two hybrid analyses (6), using SMT.

Several powerful programs exist that are helpful in analyzing and visualizing SMT data (45–47). With SMTracker (version 1), we sought to establish a GUI that allows non-specialists to obtain information on single molecule dynamics. Here, we present SMTracker 2.0, a version that contains novel tools that greatly enhance the versatility of this analytic program. These features include determination of diffusion constants via mean squared displacement (MSD), squared displacement (SQD, which can be visually represented by jump distance – JD – analysis), apparent diffusion (AppD), as well as Gaussian mixture modelling (GMM). SQD/JD, AppD and GMM can be used to determine if molecule motion indicates one or several populations of molecules with distinct diffusion constants (up to three populations can be fitted), and several statistical tests and Bayesian information criterion (BIC) are employed to avoid overfitting of data. Abundance of tracks within a standardized cell can be visualized by ‘heat maps’, and ‘speed maps’ visualize subcellular localization of populations having distinct diffusion constants. SMTracker 2.0 contains a tool for automated molecule counting, which we show performs well, especially for low-expressed proteins (14), and must be corrected because of underestimation of maximum 15% molecules per cell at high copy number (from 40 to 500). We have also developed a tool visualizing molecule tracks that remain confined by a certain number of steps within a chosen radius (which can be suggested by the program dependent on the localization error multiplied by a factor of three), of molecules showing no confinement, and molecules showing transitions between confined and freely diffusive motion. All three patterns of movement can be quantified, such that percentage of e.g. transitions between bound and diffusive motion can be determined, for example for a given molecule under normal and stress conditions, or for a protein binding to and unbinding from a protein complex. To further investigate different modes of diffusion, we have included MSD-based analyses of tracks to determine if Brownian motion, sub- or superdiffusion may underlay molecule movement, which we have e.g. employed to analyse motion of actin-like MreB protein at a single molecule level (15). A further handy tool we have developed is the ability to study proximity of proteins and defined subcellular sites. For example, we have used this tool to show that DNA polymerase A (analogue of DNA Pol I in *E. coli*) is rarely present at the replication fork, and is recruited to this multiprotein complex upon induction of DNA damage (17).

Using SMTracker 2.0, we show that the proposed RNA degradosome centered around RNase Y may consist of RNase Y and enolase, and helicase CshA that we have not investigated here, and may contain molecules of RNase J1 and J2, or of PfkA, but likely only in a substoichiometric amount and/or highly transient manner. The latter molecules might interact in a low-affinity manner, e.g. when they find substrate at the RNase Y/enolase structures, but based on a very different behaviour at a single molecule level are not stoichiometric complex partners. We show that RNase Y is present at an average copy number of about 500 (after correction by 15% for underperformance above 20 molecules per cell, and additionally for observed dimerization) molecules per cell, comparable to the number determined by mass spectrometry analyses (38). We show that about 46% of RNase Y molecules are statically positioned, e.g. likely in a substrate/degradosome-bound state, and thus, about 223 molecules of RNase Y are in this state on average. Corroborating *in vitro* data (39), we provide evidence suggesting that RNase Y forms dimers *in vivo*, based on the detection of single particles containing twice the fluorescence intensity of single RNase Y molecules, which bleach in a single step. Assuming that RNase Y operates as a dimer within the RNA degradosome, our data suggest that there are about 112 of such structures on average in a growing cell, or fewer containing more than RNase Y dimers). Given the abundance of mRNA within growing cells, these structures must be highly efficient to ensure timely degradation of mRNA.

RNase Y shows at least two diffusive populations: a low mobile/static fraction and a mobile/diffusive fraction, which also holds true for the other degradosome components. RNase Y and enolase become much more mobile in response to tuning down of transcription, suggesting that in the absence of substrate, they lose static motion within the degradosomes. This behaviour was not found for RNase J1 and J2, nor for PfkA. However, we observed that in the absence of PfkA or of RNase J2, the mobility of RNase Y molecules is considerably altered, in that they become more static. These data suggest that RNase J2 and PfkA may affect the efficiency of active RNase Y molecules, or may influence RNase Y turnover in terms of unbinding and rebinding to mRNA substrate, and suggest that they do play a role within the RNA degradosome. This was supported by genetic experiments, in which cells expressing an RNaseY-mVenus fusion as sole source of the protein grow indistinguishable from cells expressing wild type RNase Y, but grow slower in the absence of PfkA or of RNase J2. These findings support the idea that PfkA and RNase J2 increase the activity of RNase Y, which in case of the fusion protein is no longer optimal in cells lacking the degradosome components (43).

Interestingly, motion at the single molecule level of RNase Y can be better explained by assuming three different populations of molecules with distinct diffusion coefficients in case of mutant cells. We suggest that this is also true for wild type cells, but is difficult to see, possibly because of transition of RNase Y between different mobility states or present in different complexes. In case of three populations, we would like to suggest that besides degradosome-engaged (static) molecules and freely diffusive molecules, the intermediate mobility state may correspond to RNase Y present within the Y-complex, which may have distinct functions from those in the degradosome, or that RNase Y can bind to mRNA within the cytosol or at the cell membrane, and guide it to the nearest degradosome or initiate a new degradosome. These findings suggest that RNase Y may have a more complex role within the cell, rather than being bound to the degradosome or not, and indicate that future experiments using single molecule microscopy in different genetic backgrounds may shed further light onto the spatiotemporal mode of mRNA decay and its regulation in bacteria.

In *E. coli* and other bacteria, RNase E forms the central part of the RNA degradosome, which contains additional RNases, an RNA helicase, and enolase (4,10,48). Our data strongly support the idea that enolase plays a similar role in *B. subtilis* and other bacteria that contain RNase Y instead of RNase E, and thus is also a moonlighting protein in *B. subtilis*, as has been proposed before based on interaction studies (6). Proposed degradosome components RNase J1 and J2, and PfkA were identified from crosslinked cell extracts, and from two hybrid analyses. Our single molecule analyses support their involvement in RNase Y activity, but suggest that they are only peripherally associated with RNase Y. It will be highly interesting how members of this obviously very dynamic machinery co-operate at a molecular level.

## DATA AVAILABILITY

The data that support the findings of this study are present in the publication; raw data are available on request from the corresponding author.

## Supplementary Material

gkab696_Supplemental_File
