# Supplementary material for: Single molecule/particle tracking analysis program SMTracker 2.0 reveals different dynamics of proteins within the RNA degradosome complex in Bacillus subtilis
Source: Nucleic Acids Res. 2021 Aug 20;49(19):e112. doi: 10.1093/nar/gkab696 (PMC8565344; doi:10.1093/nar/gkab696)
Supplement: gkab696_Supplemental_File [file gkab696_supplemental_file.pdf]

Single molecule/particle tracking analysis program SMTracker 2.0 reveals different dynamics of proteins within the RNA degradosome complex in *Bacillus subtilis*

Luis M. Oviedo-Bocanegra, Rebecca Hinrichs, Daniel Andreas Orlando Rotter, Simon Dersch and Peter L. Graumann

SYNMIKRO, LOEWE Center for Synthetic Microbiology, Marburg, Germany;
Department of Chemistry, Philipps Universität Marburg, Germany.

Supplementary Material

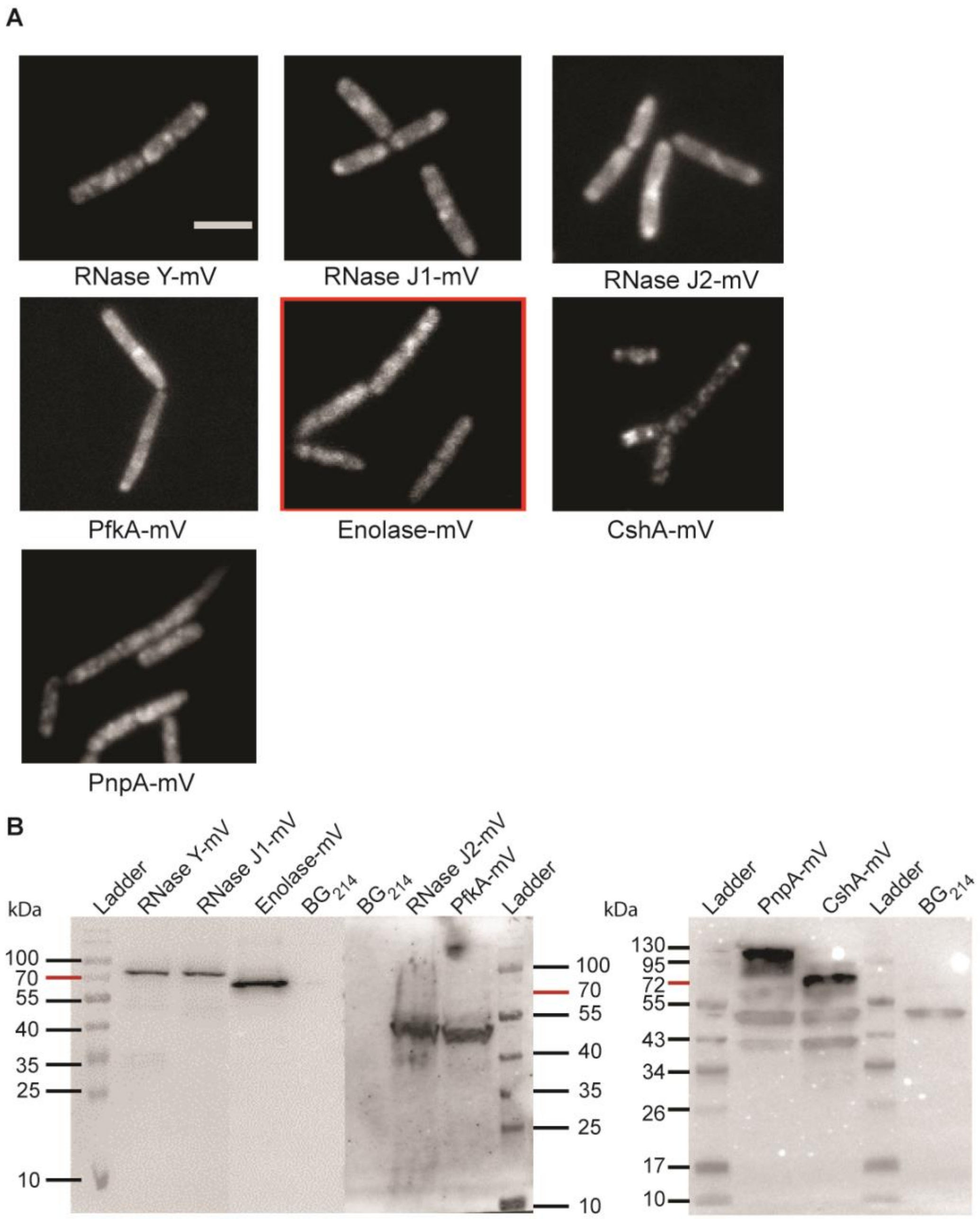

Figure S1. Localization of degradasome proteins by epifluorescence (A) RNase Y-mV, RNase J1-mV, RNase J2-mV, PfkA-mV, CshA-mV, PnpA-mV were expressed from the native locus. Enolase-mV (red frame) was ectopically induced (low expression) under a xylose promoter. Cells were incubated until mid-exponential phase. White bars 2 µm. (B) Western blots of the mVenus-fusions. Among the protein sizes, 26.9 kDa are calculated for the fluorescent protein (mVenus). RNase Y-mV (85.65 kDa), RNase J1 (88.24 kDa), RNase J2 (83.57 kDa), PfkA (61 kDa), Enolase (73.32 kDa), CshA (57.11 kDa), PnpA (77.28 kDa), BG214 wild type cells as negative control.

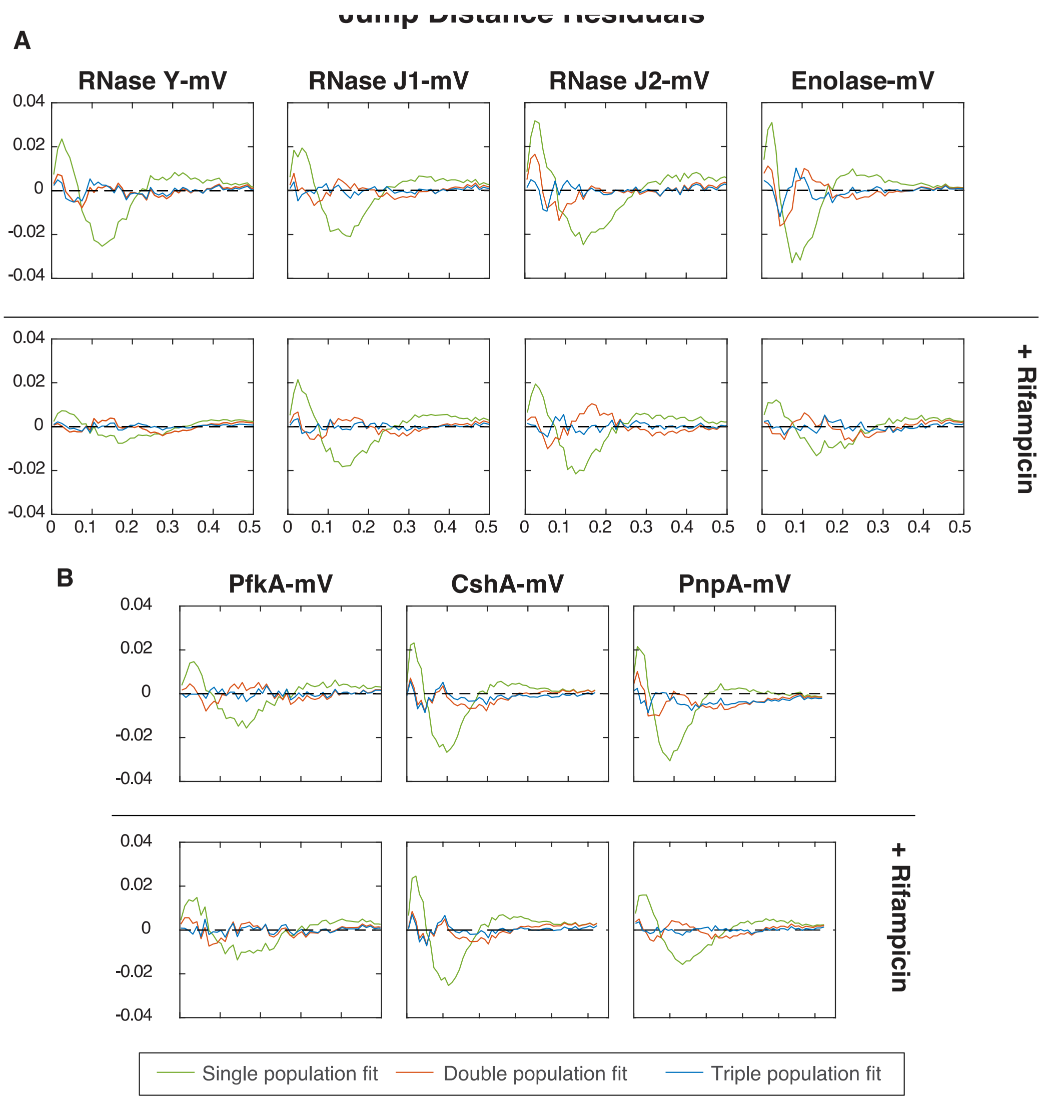

Figure S2. Residuals (difference between empirical data and the fitted model) obtained on the test dataset from the Squared Distance Analysis, for one (green), two (red) and three (blue) different diffusive populations.

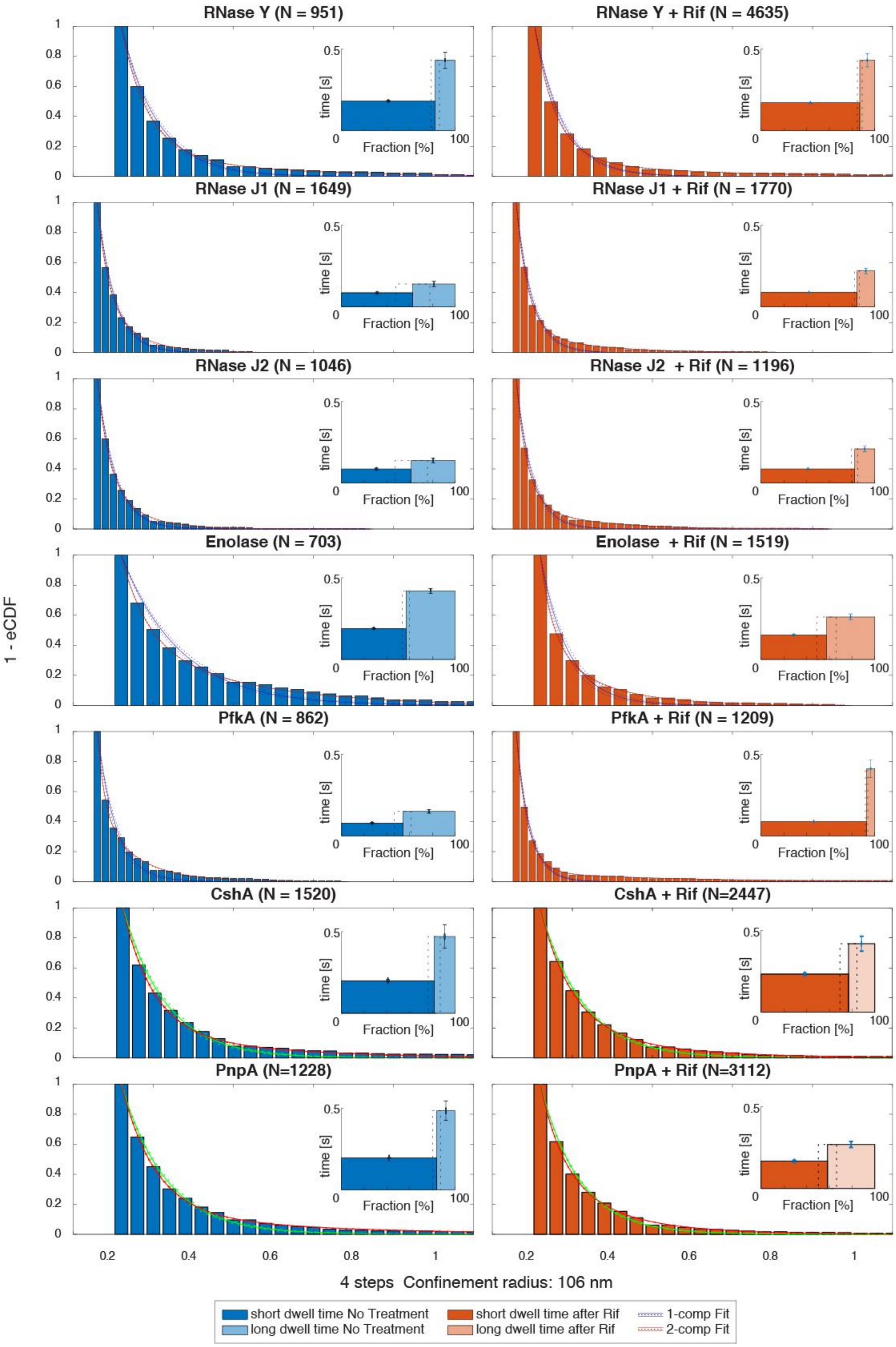

RNase Y (N = 951)
RNase Y + Rif (N = 4635)
RNase J1 (N = 1649)
RNase J1 + Rif (N = 1770)
RNase J2 (N = 1046)
RNase J2 + Rif (N = 1196)
Enolase (N = 703)
Enolase + Rif (N = 1519)
PfkA (N = 862)
PfkA + Rif (N = 1209)
CshA (N = 1520)
CshA + Rif (N=2447)
PnpA (N=1228)
PnpA + Rif (N=3112)
1 - eCDF
time [s]
Fraction [%]
4 steps Confinement radius: 106 nm
short dwell time No Treatment
long dwell time No Treatment
short dwell time after Rif
long dwell time after Rif
1-comp Fit
2-comp Fit

Figure S3 Determination of dwell times, for each protein as indicated within the panels, without and after Rifampicin treatment (in columns). Inlets show mean dwell times and proportion as a result of a double exponential decay fit to the survival function (probability of molecules confined at least a certain amount of time). Dotted areas and error bars indicate uncertainty of the parameter estimation via confidence intervals at 95%.

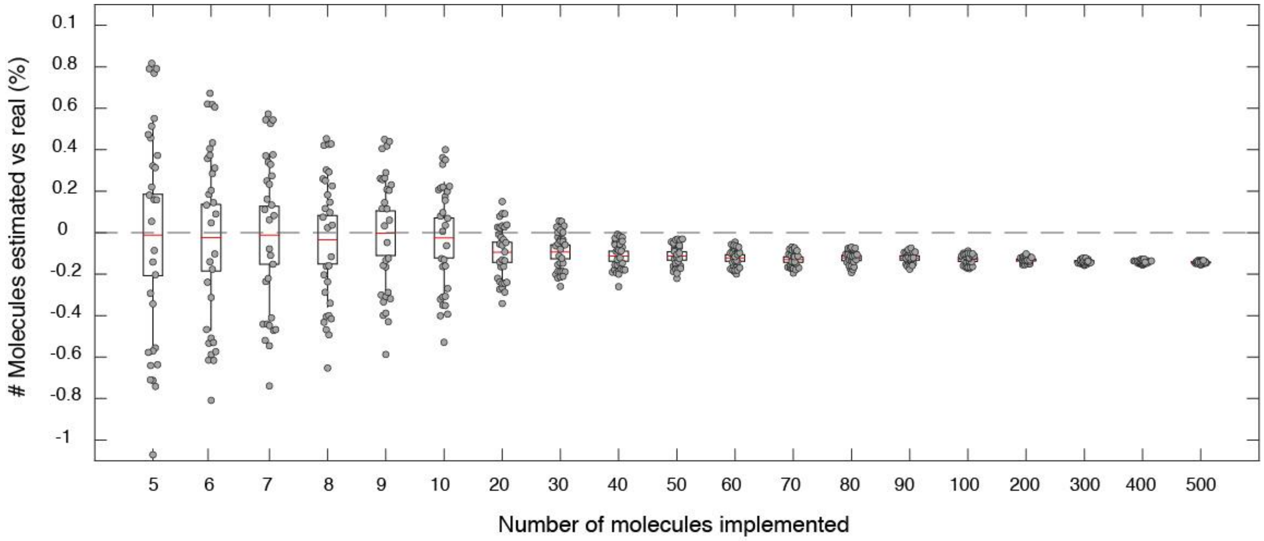

Figure S4. Simulation results for benchmarking the capabilities of the Molecule quantification tool. Shown are simulated versus estimated results and a boxplot, for which the median is marked as a red solid line. For 30 different synthetic cells, a varied number of fluorophores from 5 to 500 have been randomly inserted. The closer to 0, the better the algorithm performs. With less than 20 copies of the protein, the median remains close to 0, while a constant underfitting of 15% appears from 20 copies of the molecules, which can be used as a stable correction factor in SMTracker.

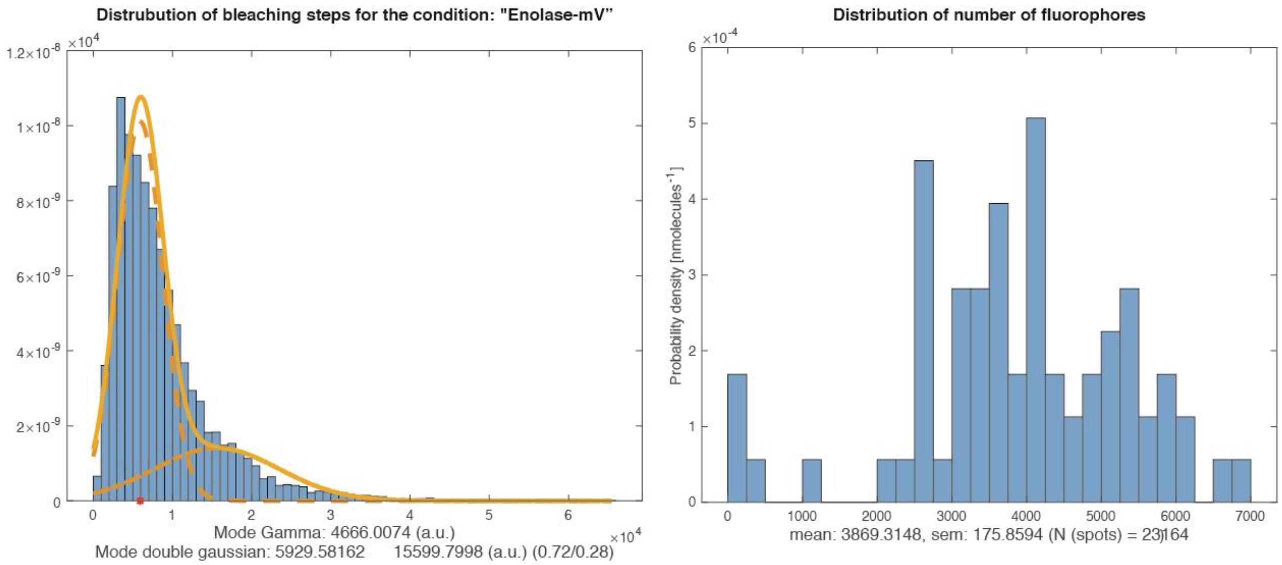

Figure S5. Automated fluorescence-based quantification of enolase molecule copy number. Left panel: distribution density function of integrated spot intensity. Distribution density function of the number of detected fluorophores in all cells. Note that a strain expressing enolase-mVenus from the original gene locus, as sole source of the protein, was used for these analyses, rather than the *amy* site-expressed fusion used for single molecule tracking experiments.

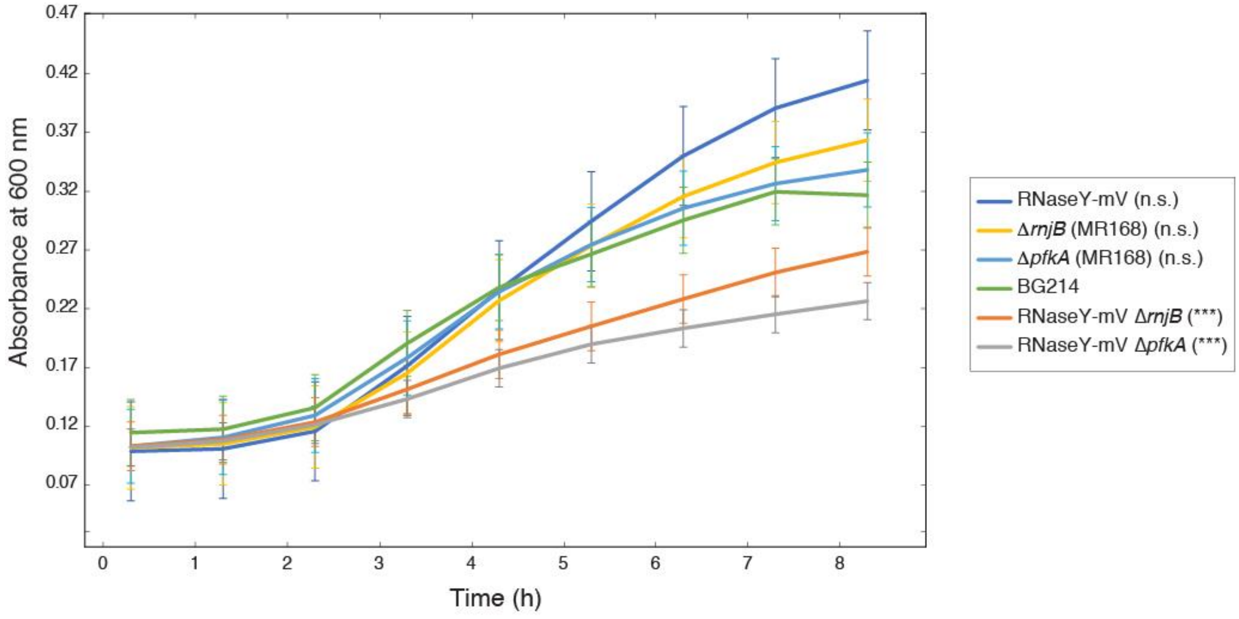

Figure S6. Growth curves for strains containing the following strains: RNase Y-mV (dark blue), RNase Y-mV Δ*rnjB* (orange, cells are lacking RNAse J2), RNase Y-mV Δ*pfkA* (grey), Δ*rnjB* (yellow), Δ*pfkA* (light blue), BG214 (green). Measurements were performed in 96-well plates with a volume of 150 µl, using a microplate reader. Differences in growth compared with the strain BG214 were statistically analysed. value: The symbols *, ** and *** represent *P*-values lower than 0.1, 0.05 and 0.01, respectively, “n.s.” statistically not significant.

Table S1 Primers used in this study

Primer	*Sequence 5´→ 3´*
PG3851 fw	AAGGAGATTCCTAGGATGGGTACCGCTTGGTGAAGACGCAA AGCT
PG3851 rev	CCTCCCAGGCCAGATAGGCCGGGCCCTTTTGCATACTCTAC GGCTCG
PG3855 fw	AAGGAGATTCCTAGGATGGGTACCGATTCACGGTGAGTACAGAATGC
PG3855 rev	CCTCCCAGGCCAGATAGGCCGGGCCCAACCTCCATAATGATCGGCA
PG3856 fw	AAGGAGATTCCTAGGATGGGTACCGGTAAACGGCGAATACAGAAT
PG3856 rev	CCTCCCAGGCCAGATAGGCCGGGCCCTACTTCCATAATAATTGGGATG ATC
PG4041 fw	AAGGAGATTCCTAGGATGGGTACCGATTCGCGATACAGCGACTT
PG4041 rev	CCTCCCAGGCCAGATAGGCCGGGCCCGATAGACAGTTCTTTTGAAAGC
PG3857 fw	CTAGAAAGGAGATTCCTAGGATGCCATACATTGTTGATGT
PG3855 rev	AGGCCAGATAGGCCGGGCCCCTTGTTTAAGTTGTAGAAAG

Table S2 Strains used in this study

Strain or Plasmid	*Relevant features*	*Reference or source*
B. subtilis		
BG214	Wild type	
PG3844	*rny*-mVenus^{cmR}	This study
PG3845	*rnjA*-mVenus^{cmR}	This study
PG3846	*rnjB*-mVenus^{cmR}	This study
PG3847	amyE::Pxyl-*eno*-mV	This study
PG3848	pfkA-mVenus^{cmR}	This study
PG3920	*ΔrnjB*::kan trpC2 *rny*-mVenus^{cmR}	This study
PG4040	*ΔpfkA*::kan trpC2 *rny*-mVenus^{cmR}	This study
E. coli		
DH5α	*sup*E44 Δ*lac*U169 φ80d*lac*ZΔM15 *hsd*R171 *rec*A1 *end*A1 *gyr*A96 *thi*-1 *rel*A1	New England Biolabs (NEB)
PG3730	DH5α pSG1164-mVenus, expression Vektor, Amp^{R}, Cm^{R}	(Lucena et al. 2018)
PG332	DH5α pSG1193-mVenus, expression Vektor, amy-locus Pxyl, Amp^{R}, Spec^{R}	(Feucht A & Lewis PJ 2001)
PG3851	DH5α pSG1164 *rny*-mVenus, expression Vektor, Amp^{R}, CM^{R}	This study
PG3855	DH5α pSG1164 *rnjA*-mVenus, expression Vektor, AmpR, CmR	This study
PG3856	DH5α pSG1164 *rnjB*-mVenus, expression Vektor, Amp^{R}, Cm^{R}	This study
PG4041	DH5α pSG1164 *pfkA*-mVenus, expression Vektor, Amp^{R}, Cm^{R}	This study
PG3857	DH5α pSG1193 eno-mVenus, expression Vektor, amy-locus Pxyl, Amp^{R}, Spec^{R}	This study

Table S3 Single molecule tracking setup parameters

Description	*Value*
Number of tracks	2000
Max number of points/track	20
Simulation time lag τ *(ms)*	0.02
Observation time lag (ms)	20
Number of simulation runs	30
Diffusion constant D_1 *(*$\mu m^2\ s^{-1}$*)*	[0.01 0.05 0.1]
Diffusion constant D_2 *(*$\mu m^2\ s^{-1}$*)*	[0.1 0.5 1]
Fraction size α *(% molecules at diffusion rate* D_1*)*	[20 40 60 80]
Localization error ξ *(nm)*	30

Table S4 Configuration of the synthetic trajectories

Description	*Value*
#Empirical movies with wt cells	10
#Empirical movies with wt cells to simulate autofluorescence	40
#Empirical movies with wt cells to simulate wild-type cells with FP	30
Sigma noise of spot fluorescence	50 a.u.
Typical fluorescent spot intensity	2500 ± 200 a.u.
#molecules per cell, Nmol	[5:9 10:10:100 200:100:1000]
Localization error	30 nm
#molecules added to simulate initial fluorescence	$n_1=0.9\cdot n_{tot}$.

Supplementary Methods

1. SMTracker software updates

1.1. Stationary Localization Analysis (SLA) panel

Residence or dwell time is defined as the average duration that a particle stays inside a certain region. Observing the trajectories in this manner could give insights, for example, on how long the degradosome is bound before disassembling mRNA. For that matter, dwell times calculations need as parameters the region, in this case a circle is the type chosen, and the minimum number of steps that should remain inside the circle (1 step = 1 interval time).

The procedure operates in such a way that searches for the longest dwell events of the protein in each trajectory (a "dwell event" occurs when a trajectory has at least one consecutive subset of nodes that fulfills the conditions of minimum number and spread). Being T = $\{C_1, \dots, C_n\}$ a trajectory defined as a set of nodes $C_i = (x_i, y_i)$, and x_i, y_i the nodes coordinates in a cartesian axis, the circle $C(C_k, R)$ is chosen, -with R being the radius- that contains the maximum number of consecutive points of the trajectory. Then, the amount of time the molecule stays is stored, and the same track T excluding that segment of trajectory , $T/\{C_k, \dots, C_{k+p}\}$, is again searched for more dwell events. The procedure finishes when no more dwell events can be found. In our procedure, one gap (point absent for one frame) or one point outside the circle that goes and comes back are also considered to have remained inside the circle (for quantification purposes). The number of dwell events and their frequency is plotted in a pdf-histogram, and this data is fitted to a multi-exponential decay

$$d(t) = \sum_{i=1}^{2} \alpha_i \cdot e^{-\frac{t-t_0}{\tau_i - t_0}} \qquad (1)$$

in order to distinguish up to two different populations of dwell times events. In another plot, it is shown the average number of dwell events per length of the track + standard error.

The results of this quantification are displayed in the table as follows:

- average dwell time (s)
- The estimated dwell time for 1 pop
- Estimated dwell times for 2 populations and their percentage.

Additionally, the result of the Kolmogorov-Smirnov hypothesis test that checks whether the dwell times distributions are different compared to other conditions would be displayed in the table.

The concept of confinement or confined event happens when a protein has restricted movement for a certain amount of time, which is important to locate areas where it is interacting or to detect possible binding partners. To this end, a confinement map has been developed using the information given by the dwell times calculation algorithm. In the same way, a trajectory is considered to present confinement when it has at least one dwell event. This confinement can be total (confined track), partial (mixed behaviour), or absent (freely diffusive). Along with the confinement map, another graph is shown that holds information about the number and probability of a track of a certain length do “transitions” (transitions are defined if a protein changes its state from confined to free and vice versa). It is considered that a track has transitioned from one state to the other if the trajectory, while not in a confined state, travelled an average step distance longer than the confinement radius for at least 3 steps (3*interval time).

1.2. Mean-square displacement (MSD) analysis panel (updated)

In order to gain a better understanding of the type of motion of a protein, an individual fitting procedure to the time-averaged MSD (TAMSD) curves has been added. Given the coordinates of a trajectory with a minimum number of time lags, the TAMSD curve is obtained and fitted via non-least squares algorithm to the equation $\mathrm{MSD(t)} \sim 2\mathrm{d} \cdot \mathrm{D}_{\alpha}\ \mathrm{t}_{\alpha}$,

where α is a value between 0 and 2. The results are shown in an auxiliary panel, highlighting the frequency of Brownian, super-diffusive or sub-diffusive trajectories, depending on the values of α. The decision is supported by a Statistical F-test for nested model to determine the type of motion.

1.3. Cluster Algorithms (Clustering) analysis panel (new)

To compare the overall behaviour of every diffusive group without any previous considerations, we implemented k-means into SMTracker, an unsupervised Machine Learning method to classify all trajectories via their TAMSD, and group them into subsets (or *clusters*) depending on their distance to the centroid of each cluster. The number of clusters is set to a maximum of 6, while the optimal number of clusters is finally set using the Calinski-Harabasz criterion. This panel would be easily expandable to different types of clustering algorithms e.g., hierarchical, DBSCAN or nearest-neighbours.

1.4. Squared displacement (SQD) analysis panel (updated)

In search of more intuitive representation of the data, SQD panel has been updated with a new way of visualizing the Square-Displacement fit. Jump-Distance histograms (Weimann et al., 2013) have been added, along with F-test to choose the optimal number of diffusive populations.

1.5. Apparent Diffusion (APPD) analysis panel (new)

From the equation MSD(t) ~ 2·d·D_{app} t, being *d* the dimensionality of the data and D_{app} the apparent diffusion obtained from the linear fit of the MSD curve, the theoretical random variable D_{app} can be explained by a Gamma Distribution

$$f(D,n) = \frac{\left(\frac{n}{D}\right)^n x^{n-1} e^{-\frac{nx}{D}}}{(n-1)!} \tag{2}$$

(Stracy et al, 2015,) and similarly by a Gaussian Distribution

$$g(x|D,\sigma) := \frac{1}{\sigma\sqrt{2\pi}}\, e^{-\frac{1}{2\left(\frac{x-D}{\sigma}\right)^2}} \tag{3}$$

(Xu, M. et al, 2019, Zhang, M. et al, 2017).

Accordingly, for multiple diffusive species with diffusion constants D_i $(i = 1, \dots, q)$ and relative fractions α_i the probability density function reads

$$P(r^2, t) = \sum_{i=1}^{q} \alpha_i \cdot h(x) \tag{4}$$

where $\sum_{i=1}^{q} \alpha_i = 1$, and $h(x) = f(x|D,n)\ or\ g(x|D,\sigma)$, in the Gamma or Gaussian Distribution case respectively. For a given number of diffusive states q, the algorithm implemented in the SQD panel performs a nonlinear least-square fit of Eq. (3) to the experimental pdfs therefore estimating the α_i and D_i. On user's request, D_i could be fixed, for each $i = 1, \dots,$ q, being $\mathrm{q} = 3$ in our setup.

1.6. Spatial distribution (SDA) panel (updated)

The spatial distribution panel summarizes the localization distributions for the detections of the fluorescent protein into a cell-centric coordinate system and then scaled to a standardized cell This tool has been improved by the inclusion of new filters according to the cell size or the type of diffusion a certain trajectory has. In addition, binned heat maps to show the dynamics of a molecule related to the local position in the cell have been included.

1.7. Distance calculator tool (new)

Determining co-localization of two interacting proteins is an interesting property that can be laborious when freely diffusive proteins is the subject of study. SMTracker includes a tool that permits visualization of such events and provides a histogram to characterize the movement of a freely diffusive protein relatively to a fixed focus. For each cell, one or more foci can be selected, and it is displayed the histogram of distances from the detected trajectories to them. If several foci are marked, the distance to the closer focus is considered.

1.8. Molecule quantification tool (new)

Knowing the copy number of a protein is extremely useful to understand the function and mechanisms of it. For this purpose, we have developed a tool that estimates the number of copies of a protein based on the Single Molecule Tracking pipeline. In short, the quantification of fluorophores has two steps: The estimation of the bleaching step of a single fluorophore (A) and the measurement of the integrated intensity of the cell right after the laser illuminates the sample (B). Dividing the latter by the former will give us a good estimation of the number of fluorophores presented inside a cell. A more detailed explanation can be found below.

A. Estimation of a single bleaching step
  a) First, after having done a regular tracking procedure with minimum length of the track of 3 frames, for every frame that holds a track, the intensity would be calculated as follows:
  b) Apply illumination correction and background subtraction to the frame.
    i) Set two masks, one circled mask centred on the spot exact location with diameter 6 pixels, and the other mask with the cell contour extended in 2 pixels. The median of the pixels outside the inner circle but inside the cell will be considered the whole contribution of background and subtracted to the intensity inside the inner circle.
    ii) The integrated intensity of the spot is the sum of intensity per pixel inside of the inner circle.
    iii) Move forward to the next frame and back to step i)
    iv) Once the last frame of this track is reached, a median filter to the intensity data vs time is applied to clean noisy data. Every resulting intensity is stored.
    v) A multiple Gaussian fit to the histogram of “spot intensities” is used to infer the intensity of a single fluorophore. The mean of the gaussian component with the lower order peak is considered as the best estimate for the unitary step size (like proposed by Badrinarayanan et al., 2012). Coffman & Wu, 2012 suggest using the mode of a Gamma Distribution.

B. Initial Integrated intensity

After background subtraction and corrected by uneven illumination and autofluorescence contribution, the average of the first two frames after the laser is on is considered.

C. Uneven Illumination correction (ratio)

In order to correct uneven illumination along the focused field, a ratio matrix has been constructed. Using movies without any cells previously corrected by instrumental background, the mean of the intensities for every pixel after the laser is on is calculated and divided by the maximum intensity. This would give for every pixel a ratio with values between 0 and 1. This matrix must divide the intensity of every frame.

D. Instrumental background subtraction (offset)

The median of the first 20 frames before the laser is on is subtracted to each frame. (offset like in Coffman et al, 2011)

E. Autofluorescence contribution

To estimate the amount of fluorescence that comes from cell autofluorescence, a quadratic regression model to the background signal and autofluorescence signal in wild type cells using several movies (typically 10) has been applied. Then implementing the same model to the movies containing cells with tagged proteins we recover the autofluorescence contribution at the moment that the laser is switched on.

F. Considering both types of correction, the measured intensity is resulting from the equation: Intensity = (Observed_intensity - offset) / ratio.

1.9. Statistical tests

Statistical tests

To validate each result, Kolmogorov-Smirnov Goodness of fit tests were performed to assess the fitting procedure. PP-plots have been used to point out graphically the goodness of fit as well. Also, Kolmogorov-Smirnov 2-test was used to stand significant

differences between 2 empirical distributions of steps distances, or squared ones, dwell times distributions and Apparent Diffusion empirical probability density values. F-test for nested models were used to avoid overfitting in the identification of anomalous tracks.

2. Benchmarking SMTracker performance with synthetic SMT microscopy movies

To validate the performance of SMTracker, we used computational simulations to generate sets of synthetic SMT movies. Applying the molecule quantification tool allows benchmarking the performance of this method, as detailed in the following.

2.1. *Simulation of synthetic single molecule images*

Synthetic movies were produced using a 16x38x2000 matrix representing a stack of images of 2000 frames, and each cell of the matrix corresponding to the fluorescent intensity, simulating the pixels. These pixel values have been randomly sampled from the empirical distribution of intensities taken from real microscopy movies, both containing no cells (only instrumental and experimental noise) and adding *Bacillus Subtilis* 3610 wild-type cells. In addition, we worked with the idea that if a molecule bound to a fluorescent protein (FP) is hit by the laser, it will produce a trajectory at least for 3 frames. Therefore, molecules were simulated with synthetic trajectories in a 1 to 1 ratio, which were inserted into a standard wild-type cell like in previous work (Roesch et al, 2018), incorporating the illumination contribution of a fluorescent spot + some noise, and convolving the signal with a Gaussian illumination profile (Zhang, B. et al, 2006).

Finally, being $n_{tot} = n_1 + n_2$ the total number of molecules simulated, the illumination coming from excited fluorescent proteins when the laser beam is emitted was reproduced; n_1 molecules were located starting in the 30th frame and the resting n_2 were located in a random frame (beyond the 500th) in the movie. The fluorophore lifetime typically follows an exponential decay distribution, which makes the behaviour of bleaching-down in a Single Particle Tracking Slim-field microscopy experiment to be reproduced.

2.2. *Benchmarking molecule quantification*

For each value N_{mol} of number of molecules, 30 synthetic cells were created to replicate a regular experiment in our lab (of 10 movies with an average of 3 cells per movie). N_{mol} is the average number of molecules simulated per cell.

After inserting our package of synthetic movies into the SMTracker pipeline, estimated vs simulated number of molecules were compared (Figure S4). The median has been chosen as the estimator of the average number of fluorophores per cell. There is a maximum of 15% of deviation from the gold truth that stays constant from approximately 20 molecules.

References

1. Stracy M., Lesterlin C, Federico Garza de Leon, Stephan Uphoff, Pawel Zawadzki, and Achillefs N. Kapanidis (2015) Live-cell superresolution microscopy reveals the organization of RNA polymerase in the bacterial nucleoid. *PNAS* **112** (32): E4390-E4399.
2. Haas, B.L., Matson, J.S., DiRita, V.J., and Biteen, J.S. (2014) Imaging live cells at the nanometer-scale with single-molecule microscopy: obstacles and achievements in experiment optimization for microbiology. *Molecules* **19**: 12116–12149.
3. Kass, R.E., and Raftery, A.E. (1995) Bayes Factors. *J. Am. Stat. Assoc.* **90**: 773-795
4. Schütz, G.J., Schindler, H., Schmidt, T. (1997) Single-molecule microscopy on model membranes reveals anomalous diffusion. *Biophys. J.* **73**:1073–80.
5. Borgmann, L.A.K., Ries, J., Ewers, H., Ulbrich, M.H., and Graumann, P.L. (2013) The bacterial SMC complex displays two distinct modes of interaction with the chromosome. *Cell Rep.* **3**: 1483–1492.
6. Jaqaman, K., Loerke, D., Mettlen, M., Kuwata, H., Grinstein, S., Schmid, S.L., and Danuser, G. (2008) Robust single-particle tracking in live-cell time-lapse sequences. *Nat. Meth.* **5**: 695–702.

7. Manzo, C., and Garcia-Parajo, M.F. (2015) A review of progress in single particle tracking: from methods to biophysical insights. *Rep. Prog. Phys.* **78**: 124601
8. Michalet, X. (2010) Mean square displacement analysis of single-particle trajectories with localization error: Brownian motion in an isotropic medium. *Phys. Rev. E* **82**: 041914
9. Paintdakhi, A., Parry, B., Campos, M., Irnov, I., Elf, J., Surovtsev, I., and Jacobs-Wagner, C. (2016) Oufti: an integrated software package for high-accuracy, high-throughput quantitative microscopy analysis. *Mol. Microbiol.* **99**: 767–777.
10. Xu M, Ross JL, Valdez L, Sen A. Direct Single Molecule Imaging of Enhanced Enzyme Diffusion. *Phys Rev Lett*. 2019;123(12):128101.
11. Zhang, M., He, K., Wu, J. *et al.* Single-molecule imaging reveals the stoichiometry change of epidermal growth factor receptor during transactivation by β_2-adrenergic receptor. *Sci. China Chem.* **60,** 1310–1317 (2017).
12. Sliusarenko, O., Heinritz, J., Emonet, T., and Jacobs-Wagner, C. (2011) High-throughput, subpixel precision analysis of bacterial morphogenesis and intracellular spatio-temporal dynamics. *Mol. Microbiol.* **80**: 612–627.
13. Tinevez, J.-Y., Perry, N., Schindelin, J., Hoopes, G.M., Reynolds, G.D., Laplantine, E., *et al.* (2017) TrackMate: An open and extensible platform for single-particle tracking. *Methods* **115**: 80–90.
14. Badrinarayanan, A. et al. In vivo Architecture and Action of Bacterial Structural Maintenance of Chromosome Proteins. Science 338, 528 (2012)
15. Coffman & Wu. Counting protein molecules using quantitative fluorescence microscopy. Cell Press. Volume 37, Issue 11, November 2012, Pages 499-506
16. Coffman VC, Wu P, Parthun MR, Wu JQ. CENP-A exceeds microtubule attachment sites in centromere clusters of both budding and fission yeast. *J Cell Biol*. 2011;195(4):563-572.
17. Zhang B, Zerubia J, Olivo-Marin JC. Gaussian approximations of fluorescence microscope point-spread function models. *Appl Opt*. 2007;46(10):1819-1829.
18. Weimann L, Ganzinger KA, McColl J, Irvine KL, Davis SJ, Gay NJ, et al. (2013) A Quantitative Comparison of Single-Dye Tracking Analysis Tools Using Monte Carlo Simulations. PLoS ONE 8(5): e64287. https://doi.org/10.1371/journal.pone.0064287
